# Validity, Accuracy, and Safety Assessment of an Aerobic Interval Training Using an App-Based Prehabilitation Program (PROTEGO MAXIMA Trial) Before Major Surgery: Prospective, Interventional Pilot Study

**DOI:** 10.2196/55298

**Published:** 2025-02-10

**Authors:** Sara Fatima Faqar Uz Zaman, Svenja Sliwinski, Lisa Mohr-Wetzel, Julia Dreilich, Natalie Filmann, Charlotte Detemble, Dora Zmuc, Felix Chun, Wojciech Derwich, Waldemar Schreiner, Wolf Bechstein, Johannes Fleckenstein, Andreas A Schnitzbauer

**Affiliations:** 1 Department of General, Visceral, Transplant and Thoracic Surgery University Hospital Frankfurt Goethe University Frankfurt Frankfurt am Main Germany; 2 Institute for Sports Sciences Goethe University Frankfurt Frankfurt am Main Germany; 3 Institute for Biostatistics and Mathematical Modelling Goethe University Frankfurt Frankfurt am Main Germany; 4 Department of Surgery, Knappschaft Clinics University Hospital Bochum Ruhr University Bochum Bochum Germany; 5 Department of Urology University Hospital Frankfurt Goethe University Frankfurt Frankfurt am Main Germany; 6 Department of Vascular Surgery University Hospital Frankfurt Goethe University Frankfurt Frankfurt am Main Germany

**Keywords:** digital health, prehab, major surgery, surgical oncology, smartwatches, safety and quality, surgery, surgical, oncology, validity, accuracy, safety management, management, aerobic, aerobic training, app, prehabilitation, pilot study, quality of life, medical device, wearable, wearables, heart rate

## Abstract

**Background:**

Major surgery is associated with significant morbidity and a reduced quality of life, particularly among older adults and individuals with frailty and impaired functional capacity. Multimodal prehabilitation can enhance functional recovery after surgery and reduce postoperative complications. Digital prehabilitation has the potential to be a resource-sparing and patient-empowering tool that improves patients’ preoperative status; however, little remains known regarding their safety and accuracy as medical devices.

**Objective:**

This study aims to test the accuracy and validity of a new software in comparison to the gold-standard electrocardiogram (ECG)-based heart rate measurement.

**Methods:**

The PROTEGO MAXIMA trial was a prospective interventional pilot trial assessing the validity, accuracy, and safety of an app-based exercise program. The Prehab App calculates a personalized, risk-stratified aerobic interval training plan based on individual risk factors and utilizes wearables to monitor heart rate. Healthy students and patients undergoing major surgery were enrolled. A structured risk assessment was conducted, followed by a 6-minute walking test and a 37-minute supervised interval session. During the exercise, patients wore app-linked wearables for heart rate and distance measurements, which were compared with standard ECG and treadmill measurements. Safety, accuracy, and usability assessments included testing alarm signals, while the occurrence of adverse events served as the primary and secondary outcome measures.

**Results:**

A total of 75 participants were included. The mean heart rate differences between wearables and standard ECG were ≤5 bpm (beats per minute) with a mean absolute percentage error of ≤5%. Regression analysis revealed a significant impact of the BMI (odds ratio 0.90, 95% CI 0.82-0.98, *P*=.02) and Timed Up and Go Test score (odds ratio 0.12, 95% CI 0.03-0.55, *P*=.006) on the accuracy of heart rate measurement; 29 (39%) patients experienced adverse events: pain (5/12, 42%), ECG electrode–related skin irritations (2/42, 17%), dizziness (2/42, 17%), shortness of breath (2/42, 17%), and fatigue (1/42, 8%). No cardiovascular or serious adverse events were reported, and no serious device deficiency was detected. There were no indications of clinically meaningful overexertion based on laboratory values measured before and after the 6-minute walking test and exercise. The differences in means and ranges were as follows: lactate (mmol/l), mean 0.04 (range –3 to 6; *P*=.47); creatinine kinase (U/l), mean 12 (range –7 to 43; *P*<.001); and sodium (mmol/l), mean –2 (range –11 to 12; *P*<.001).

**Conclusions:**

The interventional trial demonstrated the high safety of the exercise program and the accuracy of heart rate measurements using commercial wearables in patients before major surgery, paving the way for potential remote implementation in the future.

**Trial Registration:**

German Clinical Trials Register DRKS00026985; https://drks.de/search/en/trial/DRKS00026985 and European Database on Medical Devices (EUDAMED) CIV-21-07-0307311.

**International Registered Report Identifier (IRRID):**

RR2-10.1136/bmjopen-2022-069394

## Introduction

Postoperative complications occur in 15%-40% of patients undergoing major surgery, potentially resulting in life-threatening conditions, a decline in quality of life, or reduced physical functioning [1-3]. Adverse events (AEs) associated with surgical procedures impose a significant financial burden due to additional costs from intensive care treatment, reoperations, or prolonged hospital stays [4,5]. Validated assessment tools, such as the Risk Analysis Index (RAI) score, the Eastern Cooperative Oncology Group (ECOG) Performance Status, and the Timed Up and Go Test (TUG), can accurately identify patients’ individual risk factors and predict their surgical outcomes [6-9]. Importantly, the physical ability to recover from the physiological stress of surgery and the demands of an aggressive postoperative metabolism is strongly correlated with preoperative health status and physical functioning [10,11]. Accordingly, preoperative exercise interventions aim to enhance patients’ aerobic capacity and muscle strength, significantly facilitating postoperative recovery.

Prehabilitation, an emerging field in perioperative care, addresses modifiable risk factors before surgery through interventions such as exercise training, nutritional support, and psychocognitive training [11]. Notably, moderate- to high-intensity programs combining aerobic and resistance training over 3-6 weeks have been shown in recent studies to significantly enhance cardiorespiratory reserve and the capacity to adapt to physical stress [11-13].

Digital health technologies have the potential to enhance health management and facilitate the large-scale implementation of personalized prehabilitation programs in clinical settings by offering cost-effectiveness, broad population accessibility, and high adherence to short-term exercise programs [14,15]. In particular, mobile health (mHealth) apps that leverage mobile wireless technologies and integrate with wearable devices are powerful tools for achieving health objectives through self- and remote monitoring, personalized goal setting, and gamification [16,17]. Wearable devices used for monitoring heart rate and activity are increasingly evaluated in accuracy validation studies [18,19]. The Prehab App has been developed as a platform linked to a wearable device, enabling individualized, risk-adjusted, home-based aerobic exercise training as prehabilitation before major surgery. The objective of the prospective interventional PROTEGO MAXIMA trial was to evaluate the accuracy of wearable measurements and the safety of the prehabilitation program compared with certified electrocardiogram (ECG) measurements in a supervised and controlled environment. Remote, independent, interventional, and risk-based exercising represents a novel, stand-alone approach in prehabilitation, necessitating regulated testing in compliance with the Medical Device Regulation (MDR) [20], the DIN (Deutsches Institut für Normung; German Industry Norm) ISO (International Organization for Standardization) 14155 [21], and the Medical Device Law Implementation Act (Medizinproduktedurchführungsgesetz; MPDG) [22]. This approach also holds first-mover status for real-time data capture. The hypothesis was that the software would demonstrate comparable accuracy and validity in heart rate measurement during a 6-minute walking test (6MWT) and a 47-minute interval training session, matching the performance of a certified ECG, which is considered the gold standard.

## Methods

### Study Design and Participants

The prospective interventional PROTEGO MAXIMA trial was designed to evaluate the validity, accuracy, and safety of a prehabilitation app. Reporting was conducted in accordance with the STARD (Standards for Reporting of Diagnostic Accuracy; Multimedia Appendix 1) reporting guidelines [23]. The trial included healthy students and patients scheduled for elective major surgery, conducted between March 25, 2022, and September 29, 2022, at the University Hospital Frankfurt [24]. The study protocol has been previously published [25]. In brief, the trial initially recruited 10 healthy students from the Sports Institute at Goethe University Frankfurt to establish a baseline data set from healthy volunteers. In the next phase, 65 patients (aged 18 years or older, capable of understanding and performing the endurance exercise program, and scheduled for elective major surgery) were recruited at the University Hospital Frankfurt. Eligible surgeries included gastrointestinal resections; resections of the hepatobiliary pancreatic system, endocrine glands, lung, or bronchus; splenectomy; abdominal wall hernia repairs; urological or gynecologic resections; or vascular surgeries excluding cardiovascular procedures. We did not exclude patients with chronic but compensated cardiovascular and pulmonary conditions, as these represented a key focus group for assessing risk. The main exclusion criteria were a history of pregnancy or breastfeeding, acute cardiovascular disease, or any acute noncardiopulmonary disorder that could affect or be exacerbated by exercise performance. All participants provided written informed consent. As this is a first-in-human-use pilot trial, we broadened the scope of data collection to include as many scenarios as possible, ranging from healthy students with anticipated textbook outcomes to normal and high-risk patients. At this stage, there was no control group as we needed to evaluate the validity and general safety of the interventional medical device, similar to a phase I study in drug development.

### Ethical Considerations

The study adhered to the regulations of the DIN ISO 14155 [21], the MDR [20], and the MPDG [22]. The trial protocol was approved by the institutional review board of the University Hospital Frankfurt and by the Federal Institute for Pharmaceuticals and Medical Products (Bundesinstitut für Arzneimittel und Medizinprodukte; reference number 94.1.04-5660-13655) on February 7, 2022. The study was registered in the German Clinical Trial Register (DRKS00026985) on December 21, 2021, and in the European Database on Medical Devices (EUDAMED; CIV-21-07-0307311). Data from all patients were stored in an electronic case report form using the secuTrial system, a web-based data management application that complies with all required regulations. To assess the quality of the data and to audit the conduct of the trial, independent reviews were conducted by monitors following the monitoring plan of the clinical trial.

### Aerobic Interval Training Using the Prehab App

The Prehab App is developed by Capreolos GmbH, a spin-off company from Goethe University, as a medical device class IIa according to the MDR. Currently, it is not yet CE-certified. The app is linked to a wrist-worn wearable (iOS- and Google Wear OS-based hardware) through a software app, providing an individualized, digitized prehabilitation program for patients undergoing major surgery to enhance their functional capacity using aerobic endurance exercise training. Based on the patient’s risk factors (ECOG performance status, RAI score, TUG, hemoglobin values, and the presence of heart rate–changing medication), along with the resting heart rate, an individualized interval training program plan with a target heart rate range for moderate to vigorous training intensity (50%-80% of the maximum heart rate) is calculated using the Karvonen method (Multimedia Appendix 2) [26,27]. The predefined exercise program is then automatically uploaded to the patient’s smartphone and is ready for use with a provided wearable. By measuring heart rate and distance during exercise, the app provides feedback on the patient’s physical performance and potential alarms if the measured heart rate exceeds or falls below the suggested ranges (automated safety audit). Furthermore, the app used by the treating physician will be linked to the patient’s app, allowing for supervision by receiving the same alarms as defined in the safety audit and derived from DIN ISO 14971–compliant risk management [28]. After completing the individual exercises, a patient-reported outcome form will collect symptoms during the exercise, which feeds into the safety audit in case of more than 2 reported symptoms. In the final intended use, patients will perform the training 3-4 times a week, selecting the type of exercise (eg, cycling, walking, running) at any time of the day, thereby facilitating a home-based, patient-empowering environment. A 6MWT at the beginning and end of the training period will measure baseline and endpoint fitness. The Quality-of-Life Questionnaire for patients with cancer, consisting of 30 questions (QLQC30), will assess the quality of life before and at the end of the training period.

A detailed overview of the risk assessment app interface and the flow diagram of the exercise training performed under controlled conditions is provided in Multimedia Appendices 2 and 3, respectively. Eligible participants underwent a structured risk assessment and were assigned to a predefined risk score (1-6) based on the ECOG performance status (ECOG 0=0 points, ECOG 1=1 point, and ECOG >1=2 points), TUG (0-7 seconds=1 point and >7 seconds=2 points), RAI-C score (≤25=0 points and >25=2 points), and hemoglobin values (>13 g/dl=0 points and ≤13 g/dl=2 points). Then, after a baseline 6MWT, participants performed 1 one-time endurance exercise program comprising a 47-minute interval training (5 minutes of warm-up, followed by alternating 2 minutes of high-intensity intervals and 3 minutes of low-intensity active rests for a total of 37 minutes, followed by a 5-minute cool down period) on a treadmill at the Institute of Sports Science of Goethe University Frankfurt. Participants wore 1 smartwatch on each wrist (1 iOS [Apple Inc.] and 1 Android [Google LLC/Alphabet Inc.] software combination), which was linked to the app on the complementary smartphone. Four different device combinations were assessed to extend the validity of the measurements: iPhone 13 + Apple Watch 7 (Apple 13), iPhone SE + Apple Watch 3 (Apple SE), Samsung A52 + Samsung Galaxy Watch4 (Samsung A52), and Google Pixel 6 + Samsung Galaxy Watch4 (Google 6). The rationale for choosing these wearables was straightforward: we aimed to provide the 2 major platforms used in smartphones and wearable technologies. As Google Wear OS was only recently introduced, the Samsung Galaxy Watch4 was the only compatible solution at the time of development. An extension to other wearable solutions can be easily performed later. Heart rates and distances were measured at different time points (6 time points during the 6MWT and 19 time points during the interval training) by the wearables and compared with a 12-lead gold-standard ECG (Custo diagnostic; Custo med GmbH) and the treadmill measurement. Participants’ perceived intensity and pain during exercise were assessed using the Borg Scale and Visual and Numerical Analogue Scale [29]. The maximum rate of oxygen consumption (VO_2max_) in metabolic equivalent (MET) after the 6MWT was used to determine the baseline cardiorespiratory fitness [30,31]. Individual characteristics including wrist circumference, skin appendages, and skin humidity were assessed to evaluate their impact on the accuracy of measurements. As this is a fully regulated development process, the DIN ISO 13485 [28], the MDR [20], all applicable legislation such as the DIN ISO 27001 [32], and the recommendations for cybersecurity implemented by the Bundesamt für Sicherheit in der Informationstechnik (BSI [33]; Federal Office for Information Security) in Germany must be followed. This is done by a dedicated quality management program. Additionally, there is an external data safety and privacy officer within Capreolos GmbH who is responsible for monitoring this area. In brief, as the risk assessment is conducted by doctors on a separate app, all data are pseudonymized, and there are no personal data on the patient’s app. There is only a study-specific number plus a log-in so that the patient can only be depseudonymized by the clinical institutions.

### Safety Assessment of the Endurance Exercise Program

The aerobic exercise training was continuously supervised by a physician to ensure a safe environment. Secondary safety measures were based on blood samples—reported elsewhere—that were taken before and 15 minutes after the interval training (including creatine phosphokinase, creatine kinase-muscle-brain isoform, lactate dehydrogenase, glucose, sodium, potassium, chloride, C-reactive protein, blood carbon dioxide, lactate, and cell-free DNA). Moreover, the awareness of participants to specific alarm signals displayed on the wearable devices—by either color or haptic vibration—when exceeding or falling below the defined heart rate ranges was assessed. Symptoms, cardiac events, AEs, and serious AEs (SAEs) were assessed during the exercise and through structured telephone interviews or by visiting patients in the hospital (in case they had already undergone their planned surgery) on day 7 and 30 days after the exercise intervention [34].

### Statistical Analysis

The sample size calculation was based on the assumption that a maximal irrelevant mean difference of ≤10 bpm (beats per minute) is regarded as acceptable [18,35]. An acceptable error rate using the mean absolute percentage error (MAPE) was defined to be +10% or –10% according to recommendations [36,37]. Considering a power of 80%, a 1-sided α of .05, and a dropout rate of 25%, a sample size of 75 patients was required. Categorical variables were summarized as numbers (percentages), and differences between groups were compared with the *χ*^2^ test. Continuous variables were summarized with the Mann-Whitney *U* test or the Kruskal-Wallis test. Bland-Altman analysis, including calculations of mean differences and 95% limits of agreement (LoA), was used to compare heart rate and distance measurements. MAPE values were calculated as the error percentage between measurements. Outliers were not removed to prevent interference with assessing the accuracy of wearable measurements. Univariate and multivariate analyses were performed using a logistic regression model to assess the impact of different individual variables (eg, wrist circumference, skin humidity, skin appendices, risk scores) on the accuracy of heart rate measurement (odds ratio [OR] with or without 95% CI) and the impact of these variables on cardiorespiratory fitness was determined by VO_2max_. Statistical tests were 2-sided, and *P* values ≤0.05 were deemed statistically significant. Statistical analyses were performed using SPSS software (version 28.0; IBM Corp.) and R (R Studio, version 4.2.2/R Foundation, version 2022.07.2).

### Role of the Funding Source

The clinical trial was funded by the Else-Kroener-Fresenius Stiftung through the translational research program (number 2021_EKTP10). The funder had no role in study design, data collection, data analysis, data interpretation, or the writing of the manuscript.

## Results

### Baseline Data and Risk Factors

**Figure 1:**
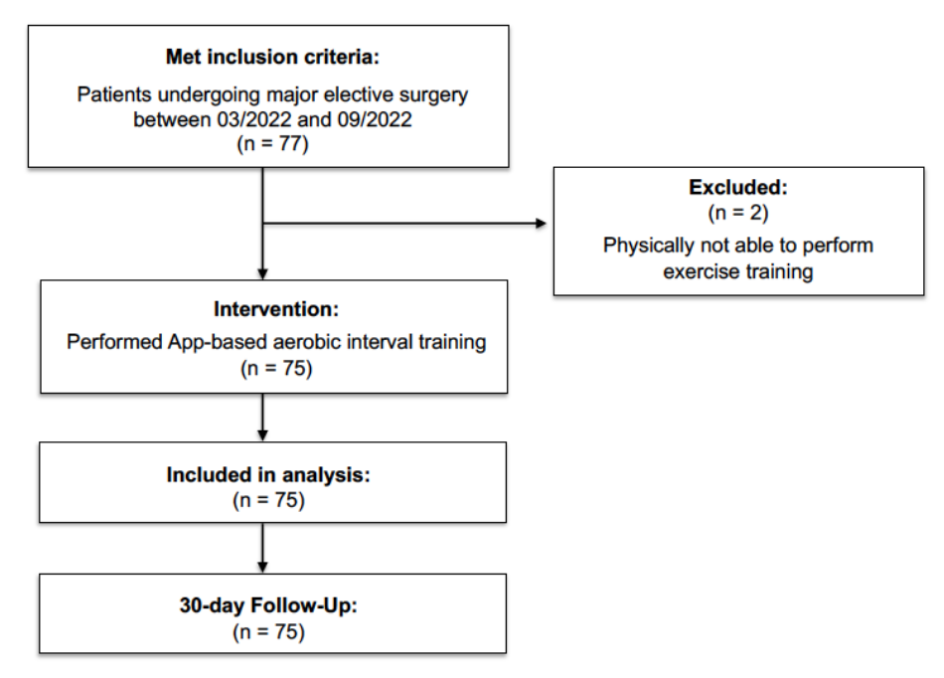
Trial profile for the interventional part of the PROTEGO MAXIMA trial: between March 25, 2022, and September 20, 2022, a total of 77 patients were enrolled, of whom 2 were excluded during exercise training. Finally, 75 patients were included in the statistical analysis.

Of the 77 participants recruited, 75 were included (Figure 1). Two individuals were excluded on the training day after double-checking the exclusion criteria because they gave misleading information during the first interview. Finally, a total of 75 participants with 150 wearables-smartphone combinations were analyzed, of whom no one was lost to follow-up. The study population was divided into 3 subgroups: 26 (35%) participants with a risk score of 0, 38 (51%) with a risk score between 1 and 3, and 11 (15%) with a risk score between 4 and 6 (Table 1). The median age was significantly higher in the risk score 4-6 group (63 vs 46 and 57, *P*=.01), consisting of significantly more male participants (9/11, 82%, for risk score 4-6, vs 12/26, 46%, for risk score 0, and 29/38, 76%, for risk score 1-3, *P*=.02; Multimedia Appendix 4 [38]). No differences were found in terms of BMI or smoking. Importantly, high- and medium-risk patients more often suffered from chronic cardiovascular or pulmonary disease compared with the other groups. High-risk patients were most commonly from the visceral (5/11, 45%) and thoracic surgical specialties (1/11, 9%) undergoing hepato-pancreato-biliary and lung resections, while urological patients (23/38, 61%) were more often assigned to the medium-risk group, scheduled for radical prostatectomy and nephrectomy. Low-risk patients predominantly comprised gynecological (4/26, 15%) and vascular surgical (3/26, 12%) patients undergoing salpingo-oophorectomy, hysterectomy, or carotid endarterectomy. Consistent with the cardiopulmonary comorbidities, the structured assessment of risk factors revealed a significantly higher percentage of RAI scores ≥26 in the high-risk group (5/11, 45%) compared with the other groups (7/38, 18%, and 0/26, 0%, for moderate- and low-risk groups, respectively; *P*<.001). Similarly, TUG scores of 7-11 seconds were more prevalent in the high-risk group (10/11, 91%) compared with the other groups (18/38, 47%, and 8/26, 31%, for moderate- and low-risk groups, respectively; *P*=.004). By contrast, no differences were observed regarding ECOG performance status and hemoglobin levels.

**Table 1:** Baseline characteristics and risk profile of the study population by app-based risk scoring.

Variables				Overall (n=75)		Risk score 0 (n=26)		Risk score 1-3 (n=38)		Risk score 4-6 (n=11)		*P* value
**Baseline characteristics**												
	Age (years); median (range)		56 (22-78)		46 (22-78)		57 (33-78)		63 (52-77)		.01	
	**Sex, n (%)**										.02	
		Female	25 (33)		14 (54)		9 (24)		2 (18)			
		Male	50 (67)		12 (46)		29 (76)		9 (82)			
	BMI (kg/m^2^), median (range)		25 (16-40)		24 (20-40)		26 (16-35)		27 (21-40)		.12	
	Smoking, n (%)		15 (20)		3 (12)		7 (18)		5 (45)		.06	
	**Medical department, n/N (%)**										N/A^a^	
		Visceral surgery	17/64 (27)		3 (12)		9 (24)		5 (45)			
		Urology	32/64 (50)		4 (15)		23 (61)		5 (45)			
		Gynecology	8/64 (13)		4 (15)		4 (11)		0 (0)			
		Thoracic surgery	3/64 (5)		1 (4)		1 (3)		1 (9)			
		Vascular surgery	4/64 (6)		3 (12)		1 (3)		0 (0)			
	**Major surgery, n/N (%)**										N/A	
		Radical prostatectomy	26/64 (41)		3/64 (5)		18/64 (28)		5/64 (8)			
		Hepatobiliary pancreatic resections	9/64 (14)		0/64 (0)		1/64 (2)		4/64 (6)			
		Partial or total nephrectomy	6/64 (9)		2/64 (3)		4/64 (6)		0/64 (0)			
		Salpingo-oophorectomy or hysterectomy	6/64 (9)		3/64 (5)		3/64 (5)		0/64 (0)			
		Carotid endarterectomy	4/64 (6)		3/64 (5)		1/64 (2)		0/64 (0)			
		Lung resection	3/64 (5)		1/64 (2)		1/64 (2)		1/64 (2)			
		Abdominal wall hernia repair	3/64 (5)		0/64 (0)		2/64 (3)		1/64 (2)			
		Other	7/64 (11)		3/64 (5)		4/64 (6)		0/64 (0)			
	**Risk group**										N/A	
		Healthy student	11 (15)		11 (42)		0 (0)		0 (0)			
		No cardiopulmonary disease	53 (71)		13 (50)		31 (82)		9 (82)			
		Chronic cardiovascular disease	7 (9)		1 (4)		5 (13)		1 (9)			
		Chronic obstructive pulmonary disease	4 (5)		1 (4)		2 (5)		1 (9)			
**Risk scoring**												
	**Risk assessment**											
		**Eastern Cooperative Oncology Group score, n (%)**										N/A
			0		73 (97)		26 (100)		36 (95)		11 (100)	
			1		2 (3)		0 (0)		2 (5)		0 (0)	
		**Risk Analysis Index score, n (%)**									<.001	
			≤15		25 (33)		19 (73)		6 (16)		0 (0)	
			16-25		38 (51)		7 (27)		25 (66)		6 (55)	
			≥26		12 (16)		0 (0)		7 (18)		5 (45)	
		**Timed Up and Go Test, seconds, n (%)**									.004	
			0-6		39 (52)		18 (69)		20 (53)		1 (9)	
			7-11		36 (48)		8 (31)		18 (47)		10 (91)	
		Hemoglobin, g/dL, median (range)	14 (10-17)		14 (13-15)		14 (11-17)		14.0 (10-16)		.48	
**Functional capacity**												
	VO_2max_^b^ (MET^c^), median (range)		3 (1-6)		3 (1-6)		3 (1-5)		2.4 (2-3)		.17	

^a^N/A: not applicable.

^b^VO_2max_: maximum rate of oxygen consumption.

^c^MET: metabolic equivalent.

Furthermore, by analyzing individual factors that impacted VO_2max_, we found that age was a significant factor (*P*=.03) for cardiorespiratory fitness in the univariate analysis (see Multimedia Appendix 5). In general, the median VO_2max_ (in MET) for the 6MWT in all patients was 3.1 (range 1.2-6.0), indicating an overall low to moderate aerobic capacity.

### Heart Rate Measurement Accuracy

**Figure 2:**
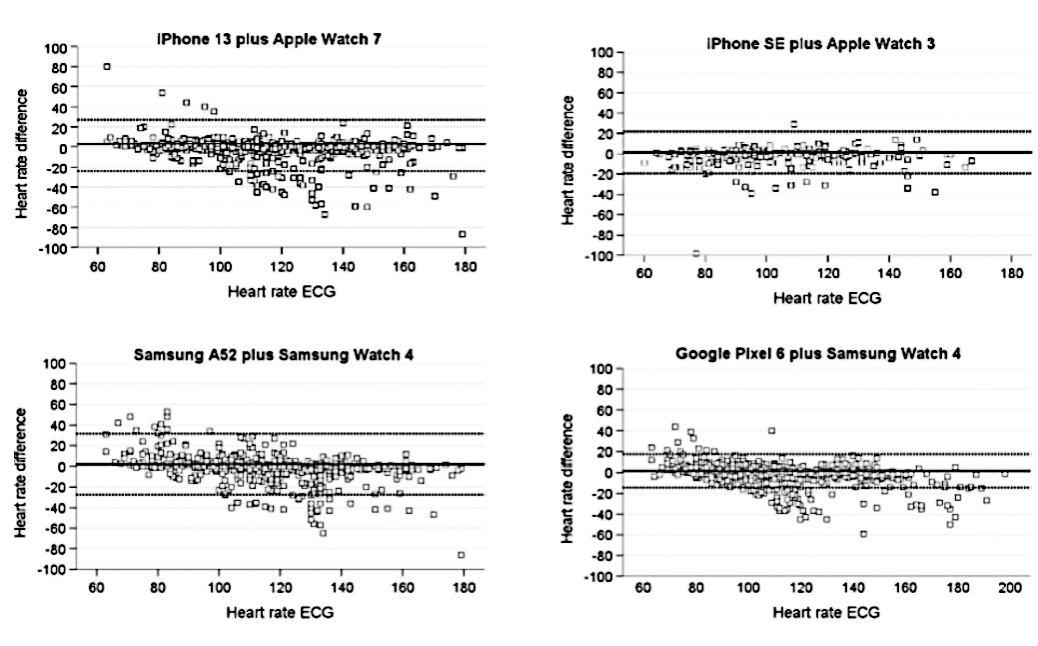
Bland-Altman plots for wearable measurement versus standard measurement: Bland-Altman plots for wearable heart rate measurements compared with electrocardiogram (ECG) measurements for 4 device combinations. The ECG heart rate, used as the reference, is shown on the x-axis, and the specified differences between ECG and the device are shown on the y-axis. The bold line corresponds to the mean difference, and the dashed lines indicate the 95% limits of agreement.

The accuracy of the heart rate and distance measurement of The Prehab App software in combination with the wearables was compared with the measurement of the standard ECG and treadmill (Table 2). The mean heart rate differences for all devices were ≤5 bpm, ranging from 2.92 bpm (iPhone SE, LoA –21.0 to 26.8) to 4.48 bpm (Samsung A52, LoA –19.1 to 27.9) for the 6MWT and from 1.33 bpm (iPhone SE, LoA –19.3 to 22.0) to 2.61 bpm (iPhone 13, LoA –22.0 to 27.1) for the interval training. Consistently, MAPE values for all 4 devices were below the threshold of 10%, with lower values for the interval training (–0.3% [Google Pixel 6] to –1.8% [iPhone 13]) compared with the 6MWT (–1.6% [iPhone 13] to –2.9% [Samsung A52]; see Multimedia Appendix 6). Results showed MAPE values well below 5% and a mean heart rate difference under 5 bpm, indicating excellent reliability of iOS and Android heart rate measurements for The Prehab App software. Bland-Altman plots with 95% LoA are depicted in Figures 2 and 2. Interestingly, all 4 wearables exhibited a slight tendency to underestimate heart rates, which was generally greater for heart rate values over 100 bpm.

For a more detailed analysis regarding the accuracy of heart rate measurement, we subsequently assessed the impact of different individual characteristics, which was defined as an MAPE of <5% (Table 3). Multivariable analysis of individual variables assessed before the exercise training indicated that a low BMI (kg/m^2^) (OR 0.897, 95% CI 0.822-0.980, *P*=.02) and a low TUG score (OR 0.122, 95% CI 0.027-0.546, *P*=.006) were independent predictive factors for high accuracy of the heart rate measurement.

**Table 2:** Comparison of heart rate and distance measurement between smartwatch versus ECG and treadmill.

Variables					iOS devices						Android devices		
					iPhone 13 + App Watch 7 (n=38)			iPhone SE + App Watch 3 (n=36)			Samsung A52 + Samsung Galaxy Watch4 (n=34)		Google Pixel 6 + Samsung Galaxy Watch4 (n=42)
**Heart rate measurement**													
	**6-minute walking test**												
		Mean (SD) (beats per minute)	105 (20)			101 (18)			106 (19)			100 (18)	
		Mean difference (beats per minute)	2.98			2.92			4.48			3.50	
		MAPE^a^ (%)	–1.62			–1.76			–2.87			–2.24	
		LoA^b^ (lower to upper; beats per minute)	–23.9 to 29.9			–21.0 to 26.8			–19.1 to 27.9			–21.1 to 28.1	
	**Interval training**												
		Mean (SD) (beats per minute)	111 (23)			112 (26)			112 (21)			111 (23)	
		Mean difference (beats per minute)	2.61			1.33			2.16			1.54	
		MAPE (%)	–1.80			0.53			0.45			0.30	
		LoA (lower to upper; beats per minute)	–22.0 to 27.1			–19.3 to 22.0			–27.4 to 31.7			–19.6 to 22.7	
**Distance measurement**													
	**6-minute walking test**												
		Mean (SD) (m)	494 (165)			537 (163)			519 (181)			510 (169)	
		Mean difference (m)	–29.22			–104.28			–31.67			–50.57	
		MAPE (%)	14.71			30.61			15.28			20.34	
		LoA (lower to upper; m)	–325.6 to 267.2			–361.8 to 153.2			–411.6 to 348.3			–373.1 to 272.0	
	**Interval training**												
		Mean (SD) (m)	3650 (1596)			3813 (1327)			4069 (1562)			3612 (1517)	
		Mean difference (m)	27.51			–24.15			–12.27			208.15	
		MAPE (%)	0.05			–0.58			3.25			–6.95	
		LoA (lower to upper; m)	–2393.1 to 2448.1			–1824.4 to 1776.1			–2658.6 to 2634.0			–2003.6 to 2420.0	

^a^MAPE: mean absolute percentage error.

^b^LoA: limits of agreement.

**Figure 3:**
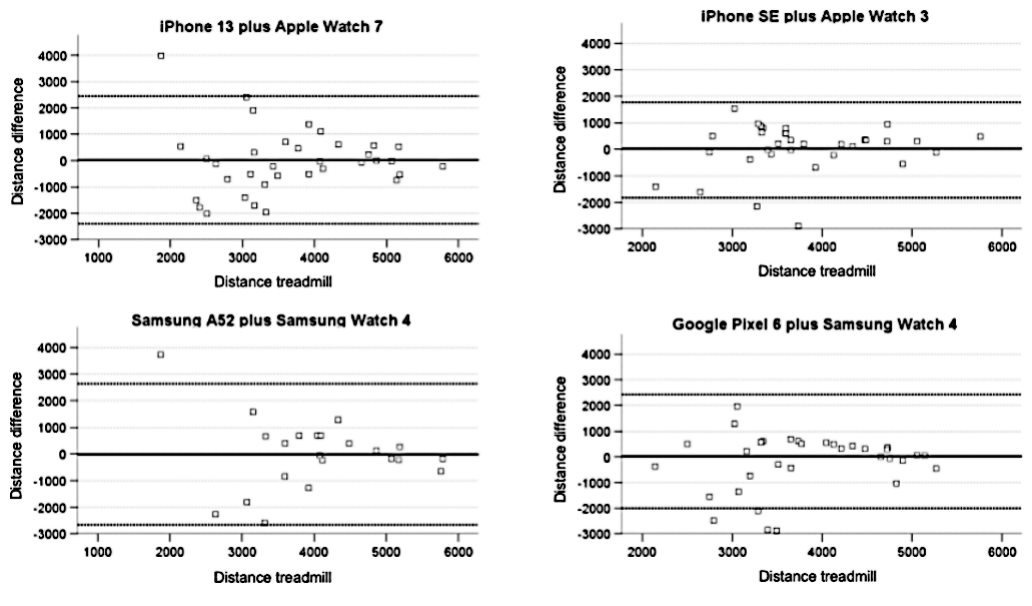
Bland-Altman plots for wearable measurement versus standard measurement: Bland-Altman plots for wearable distance measurements compared with treadmill measurements for 4 device combinations are depicted.

**Table 3:** Univariate and multivariate analyses for accuracy of heart rate measurement.

Variables						Univariate analysis				Multivariate analysis	
				Wearables (n=150)		Odds ratio (95% CI)		*P* value	Odds ratio (95% CI)		*P* value
**Baseline characteristics**											
	Age (years), median (range)		56 (22-78)		0.97 (0.94-0.99)		.03		N/A^a^		N/A
	**Sex, n (%)**						.58		N/A		N/A
		Female	50 (33)		1.26 (0.55-2.90)						
		Male	100 (67)		Reference						
	BMI (kg/m^2^), median (range)		25 (16-40)		0.91 (0.84-0.99)		.03		0.90 (0.82-0.98)		.02
**Risk assessment**											
	**Risk Analysis Index score, n (%)**						.22		N/A		N/A
		≤15	50 (33)		Reference						
		16-25	76 (51)		0.88 (0.39-2.11)						
		≥26	24 (16)		3.74 (0.71-16.97)						
	**Timed Up and Go Test (seconds), n (%)**						.009		N/A		.006
		0-6	38 (25)		Reference		N/A		Reference		N/A
		7-11	108 (72)		0.14 (0.03-0.61)		N/A		0.12 (0.03-0.55)		N/A
	Hemoglobin (g/dl), median (range)		14 (9)		0.95 (0.73-1.24)		.70		N/A		N/A
**Individual characteristics**											
	Resting heart rate^b^ (bpm), median (range)		72 (53-118)		1.02 (0.99-1.05)		.30		N/A		N/A
	Wrist circumference (cm), median (range)		17 (14-21)		0.77 (0.60-0.99)		.048		N/A		N/A
	**Skin humidity, n (%)**						.08		N/A		N/A
		Dry	90 (60)		Reference						
		Humid	60 (40)		0.51 (0.23-1.10)						
	**Skin appendices, n (%)**						.55		N/A		N/A
		Blank	42 (28)		Reference						
		Some hair	78 (52)		0.84 (0.33-2.16)						
		Very hairy	30 (20)		0.55 (0.18-1.64)						
	Borg scale, median (range)		10 (6-15)		0.82 (0.65-1.05)		.11		N/A		N/A
	Numeric Rating Scale, median (range)		0 (0-9)		1.65 (0.80-3.41)		.18		N/A		N/A
	VO_2max_^c^ (MET^d^), median (range)		3 (1-6)		1.54 (0.94-2.54)		.09		N/A		N/A

^a^N/A: not applicable.

^b^Accuracy of heart rate was defined as a mean absolute percentage error <5%.

^c^VO_2max_: maximum rate of oxygen consumption.

^d^MET: metabolic equivalent.

### Distance Measurements and Accuracy

Distance measurements of wearables compared with the treadmill revealed a higher variation, with mean differences ranging from –29.22 m (iPhone 13) to –104.28 m (iPhone SE) for the 6MWT (–31.67 m for Samsung A52 and –50.57 m for Google Pixel 6) and from –12.27 m (Samsung A52) to 208.15 m (Google Pixel 6) for the interval training (27.51 m for iPhone 13 and –24.15 m for iPhone SE). Consistently, the LoA were generally higher, with MAPE values exceeding 10% and a peak value of 30.61% for the iPhone SE.

### Safety and Laboratory Assessments

The safety analysis of laboratory values, alarm settings of the app, and occurrence of AEs was conducted (Table 4). Lab analysis did not reveal any significant deviations in blood parameters, which remained within clinical limits. A detailed analysis from an exercise physiologist’s view will be reported elsewhere. Awareness of alarm signals displayed on the wearables during the interval training was realized with a mean of 13.5 (SD 6.2) seconds. In total, 29 of 75 (39%) experienced an AE, of which 12 (16%) occurred during the interval training, comprising pain (5, 42%), skin irritation through ECG electrodes (redness or vesicles; 2, 17%), dizziness (2, 17%), shortness of breath (2, 17%), and fatigue (1, 8%). During the follow-up at day 7, 3 of 15 (20%) participants still reported pain and 12 of 15 (80%) indicated skin irritations, which remained until the follow-up at day 30 in 2 cases. Notably, 14 events were related to the certified ECG (electrodes); however, these had nothing to do with the use of the software but had to be reported for regulatory reasons. There were no SAEs or device deficiencies that put the patient at an unacceptable risk. All AEs were periodically reported to the Bundesinstitut für Arzneimittel und Medizinprodukte according to the regulations of the MDR and the MPDG, and no AE lasted longer than 30 days. No SAE was reported during the trial, and no patient died until follow-up.

**Table 4:** Safety assessment of the app-based aerobic exercise training.

Variables			Overall (n=75)	*P* value
**Laboratory values, differences median (range)^a^**				
	C-reactive protein (mg/dl)		0 (–2 to 0.2)	.41
	Creatine kinase (U/l)		12 (–7 to 43)	<.001
	Creatine kinase-muscle-brain isoform (U/l)		0 (–4 to 5)	.06
	Lactate dehydrogenase (U/l)		12 (–132 to 69)	<.001
	Glucose (mg/dl)		–4.5 (–121 to 26)	<.001
	Sodium (mmol/l)		–2 (–11 to 12)	<.001
	Potassium (mmol/l)		0 (–0.6 to 0.6)	.997
	Chloride (mmol/l)		–2 (–9 to 9)	.01
	Bicarbonate (mmol/l)		–0.35 (–11 to 7)	.29
	Lactate (mmol/l)		0.04 (–3 to 7)	.47
**Alarm setting of wearables**				
	Time of awareness seconds, mean (SD)		14 (6)	N/A^b^
**Adverse events and overall survival, n (%)**				
	Overall adverse events		29 (39)	N/A
	**During interval training**			
		All events	12 (16)	N/A
		Skin irritation through electrocardiogram electrodes	2 (17)	N/A
		Pain	5 (42)	N/A
		Dizziness	2 (17)	N/A
		Shortness of breath	2 (17)	N/A
		Fatigue	1 (8)	N/A
	**Day 7**			
		All events	15 (20)	N/A
		Skin irritation through electrocardiogram electrodes	12 (80)	N/A
		Pain	3 (20)	N/A
	**Day 30, n (%)**			
		All events	2 (3)	N/A
		Skin irritation through electrocardiogram electrodes	2 (100)	N/A
		Serious adverse event	0 (0)	N/A
	Overall survival, n (%)		75 (100)	N/A

^a^Differences were calculated for blood samples taken before and after interval training (after-before).

^b^N/A: not applicable.

## Discussion

### Principal Findings

To the best of our knowledge, this is the first prospective trial report assessing the validity and safety of a risk-adjusted, app-based prehabilitation program using wearables for heart rate and physical activity monitoring. The analysis revealed a high accuracy of wearable heart rate measurement with a mean difference of ≤5 bpm and an MAPE of ≤5%, while distance measurement exhibited larger variations (mean difference ≤209 m and MAPE ≤31%), which can be explained through an impaired detection of patient’s wrist movement during the exercise training on the treadmill and the lack of global positioning system trackers.

Prehabilitation in this context revealed a tremendous potential for improving modifiable factors in patients and thus increasing the safety and quality of surgical care. Perry et al [39] included 178 trials of heterogeneous quality on prehabilitation for patients undergoing major abdominal surgery, reporting prehabilitation to be superior to standard care in both length of hospital stay (1.81 days vs usual care) and postoperative pulmonary complications (risk ratio 0.55). This is consistent with the findings of the RCT by Barberan-Garcia et al [13] for postsurgical complications (31% vs 62%) in a cohort of 144 high-risk patients and with the meta-analysis of Moran et al [40] (OR 0.59, *P*=.03). Importantly, the study by Molenaar et al [12], which involved 251 patients undergoing resection of colorectal cancer, reported the benefit of a 4-week in-hospital multimodal prehabilitation program with significantly reduced severe postoperative complications (Comprehensive Complication Index>20: 17.1% vs 29.7%), medical complications (15.4% vs 27.3%), and enhanced functional capacity. At this stage, it seems that a resource-intensive prehabilitation program only works in clinical trials due to the enormous infrastructure requirements and costs associated with it, including patient travel over weeks, which seems hardly feasible. This suggests a real opportunity for a well-guided and safe remote approach that additionally fosters patient empowerment [41,42].

Studies on costs also suggest that an improvement in surgical outcomes, particularly the reduction in hospital length of stay, results in a significant reduction in hospital expenditures and the opportunity cost of surgery [4,5]. A more recent meta-analysis by Punnoose et al [43] of patients undergoing orthopedic surgery found a significant impact of prehabilitation on preoperative function, muscle strength, and reduction of pain. However, the current evidence on prehabilitation remains limited, characterized by broad heterogeneity between studies and a high risk of bias. Particularly, exercise regimens were not consistently reported in terms of the individual content of the exercises (ie, training type, intensity, duration) and a lack of compliance to high-intensity training substantially impairs the implementation of prehabilitation in clinical practice [11,15].

Recent studies evaluating digital prehabilitation models provide increasing evidence for their beneficial effects compared with conventional hospital-based in-person programs [14,15,44,45]. Moorthy et al [45] assessed the feasibility of a home-based supervised prehabilitation program involving 41 patients scheduled for esophago-gastric cancer surgery, revealing a higher program completion in the digital arm compared with the in-person cohort (84% vs 14%). Further, patients using the digital service exhibited increased cardiorespiratory fitness measured by the 30-second sit-to-stand test (*P*=.02) and the median heart rate recovery (10.5-15.5 bpm; *P*=.04). Similar positive effects on patients’ adherence to digital exercise programs were found by Kadiri et al [46], who evaluated the efficacy of the “Fit 4 Surgery” app, which included 10 exercises for patients undergoing lung cancer surgery. Patients in the app cohort completed more exercise sessions (9 vs 2) in a shorter time (24 vs 45 days) before surgery and even during the postsurgical period (2 vs 0), compared with those attending hospital- or community-based classes. Importantly, a quality improvement study by Kimura et al [47] that assessed the impact of an online app-based prehabilitation program (SeamlessMD) on surgical outcomes after colorectal surgery demonstrated a significantly shorter length of hospital stay (adjusted estimates –1.15; *P*=.03), while there were no differences in terms of complication rates.

A remote prehabilitation program will empower patients’ autonomy, alleviate pressure on health care workers and infrastructure, and eliminate unnecessary travel for patients. An oncologic diagnosis, coupled with the clear short-term goal of becoming the best version of oneself before a major surgical procedure, serves as a compelling motivator for patients and is a key driver of compliance and adherence to digital therapeutics [11]. Prehabilitation models incorporating remote monitoring with wearables for heart rate, physical activity, and progress tracking have been shown to support personalized goal setting, ensure safety through professional supervision, and enhance patient adherence to the program [15,17]. Various studies have assessed wearable heart rate accuracy by comparing them with the gold-standard reference method of ECG, indicating a low overall error, low mean difference SD, and acceptable agreement with ECG for consumer wearables (eg, Apple Watch or Fitbit) [18,48-50]. Wang et al [35] assessed the accuracy of 4 different wrist-worn wearables in 50 healthy students, showing median differences of ≤10 bpm, which were generally lower at rest compared with measurements during exercise. However, despite the acceptable accuracy in laboratory settings, real-time measurement in medical settings, including an assessment of Apple Watch 3 and Fitbit Charge 2 over 24 hours compared with ECG by Nelson et al [36], indicated that single heart rate observation could be inaccurate by significantly large margins, although aggregated values revealed high overall accuracy (<10% MAPE) [51].

The study has both strengths and weaknesses that will be addressed in the following. One of the study’s strengths is that it follows a strict regulatory framework, supported by dedicated quality and risk management. All criteria assessed here were derived from risk management principles, explaining the stepwise approach that began with a first-in-human pilot study involving healthy students and patients within the intended use. The study was split into a pure usability part and an evaluation of the risk assessment, which will be reported elsewhere [26]. Another strength is the use of evidence-based risk calculation tools. The RAI score emerged as the most suitable and user-friendly score, not requiring additional tools for assessment. It, combined with the TUG and ECOG scores, best met the requirements of a digital solution. Moreover, it is currently highlighted as the so-called surgical pause by the American College of Surgeons [52]. Other scores, such as the National Surgical Quality Improvement Program (NSQIP), require significantly more resources and have not demonstrated superiority in assessments, for example, within a German health care setting [53,54].

A weakness of the study may be that it is not a randomized controlled trial. However, given that we are establishing evidence in a young field of clinical medicine, safety and validity were the first steps in the stepwise approach. This was followed by a multicentric clinical trial that is currently underway. Comparability at this stage of remote prehabilitation is challenging due to the variety of methods used to implement it. van der Velde et al [55] performed a pilot randomized controlled trial in which they used a smartphone-based app to achieve behavioral changes before surgery. They found self-reported changes in alcohol use, nicotine cessation, and an increase in self-reported exercising. Wang and coworkers [14] developed an app to foster prehabilitation and assessed its usability. While they found promising usability results with their device, no efficacy data have been presented so far. Although the ideas of the 2 previously mentioned studies were only somewhat comparable to ours, the solutions lacked supervision of the exercises, making it unclear whether the patients engaged in light or moderate to vigorous exercise, which has significantly larger effects. Haveman et al [56] followed a similar approach to ours but differed by tracking individuals’ activities and interpreting increases in heart rate and step counts 3 days before surgery as indicators of increased physical activity, without obtaining a specific readout from the assessed data. This shows that remote prehabilitation seems feasible, is being explored, and may be a hot topic for the next few years, as outlined in various arguments and discussion points previously [57].

Another challenge and critical endpoint measurement is compliance and adherence to the program provided. In this feasibility study and first-in-human use of a newly designed medical device, compliance was not an endpoint. However, the development of The Prehab App involved patients, doctors, and other experts from the very beginning [41]. The program was designed in a way that allows different risk groups to train within a general interval training framework, respecting their individual risk and adapting the individual thresholds of exercising to their profile [11,25,26]. During the randomized controlled trial, the Prehab App will reliably measure compliance using a traffic light system, as it documents every exercise in real time and provides precise information on how patients trained within their thresholds. Additionally, we will assess if the participants performed their exercises and if they took their supplementary nutrition using patient-reported outcomes. Reminders will be used to increase compliance and adherence.

All this together highlights the potential of digital prehabilitation interventions in achieving high levels of patient engagement, uptake, adherence, and usability satisfaction. App-based models provide a promising solution to overcome the limitations in scalability and sustainability seen with face-to-face programs in clinical practice. However, the application of digital prehabilitation models needs to be further assessed in multicenter randomized controlled settings to evaluate their effectiveness, particularly in terms of safety and efficacy in the final intended use, and to gain approval as a safe medical device [51,58].

### Conclusions

This analysis presents the first pilot study assessing the validity and safety of a risk-stratified prehabilitation program for patients undergoing major elective surgery, utilizing an mHealth app linked to wearable devices for self- and remote monitoring of physical performance. The heart rate measurement was below the recommended threshold with an MAPE of ≤5%, demonstrating excellent accuracy. Laboratory analysis and assessment of AEs indicated that individually tailored exercise training can be safely performed in a supervised setting, justifying the remote testing of The Prehab App in a multicentric randomized controlled trial.

### Use of Artificial Intelligence

No artificial intelligence has been used in the generation of the manuscript or any text presented here.

## Data Availability

Data can be accessed anonymously upon specific request and submission of the requesting group’s research question outline.

## Appendix

Multimedia Appendix 1 The STARD (Standards for Reporting of Diagnostic Accuracy) checklist.

Multimedia Appendix 2 Display overview of the Patronus Prehab App: (A) overview of the structured risk assessment; (B) overview of the aerobic interval training.

Multimedia Appendix 3 Flow diagram of the interventional PROTEGO MAXIMA trial. Structured risk assessment of patients undergoing major elective surgery using the Prehab App and calculation of an individual exercise program. 6MWT and interval training are performed on a treadmill in a supervised setting being connected to an ECG and wearing wearable devices. A follow-up after surgery is performed on days 7 and 30. 6MWT: 6-minute walking test; ECOG: Eastern Cooperative Oncology Group; Hb: hemoglobin; RAI: Risk Analysis Index; TUG: Timed Up and Go-Test.

Multimedia Appendix 4 CONSORT-eHEALTH checklist (V 1.6.1).

Multimedia Appendix 5 Comparison of heart rate and distance measurement between smartwatch versus electrocardiogram (ECG) according to healthy students and patients.

Multimedia Appendix 6

## Abbreviations

6MWT: 6-minute walking test
AE: adverse event
bpm: beats per minute
BSI: Bundesamt für Sicherheit in der Informationstechnik
DIN: Deutsches Institut für Normung
ECG: electrocardiogram
ECOG: Eastern Cooperative Oncology Group
ISO: International Organization for Standardization
LoA: limits of agreement
MAPE: mean absolute percentage error
MDR: Medical Device Regulation
MET: metabolic equivalent
MPDG: Medizinproduktedurchführungsgesetz
NSQIP: National Surgical Quality Improvement Program
QLQC30: 30-item Quality-of-Life Questionnaire
RAI: Risk Analysis Index
SAE: serious adverse event
STARD: Standards for Reporting of Diagnostic Accuracy
TUG: Timed Up and Go Test
VO2max: maximum rate of oxygen consumption
