# Supplementary figures and images for: Validity, Accuracy, and Safety Assessment of an Aerobic Interval Training Using an App-Based Prehabilitation Program (PROTEGO MAXIMA Trial) Before Major Surgery: Prospective, Interventional Pilot Study
Source: JMIR Mhealth Uhealth. 2025 Feb 10;13:e55298. doi: 10.2196/55298 (PMC11851035; doi:10.2196/55298)

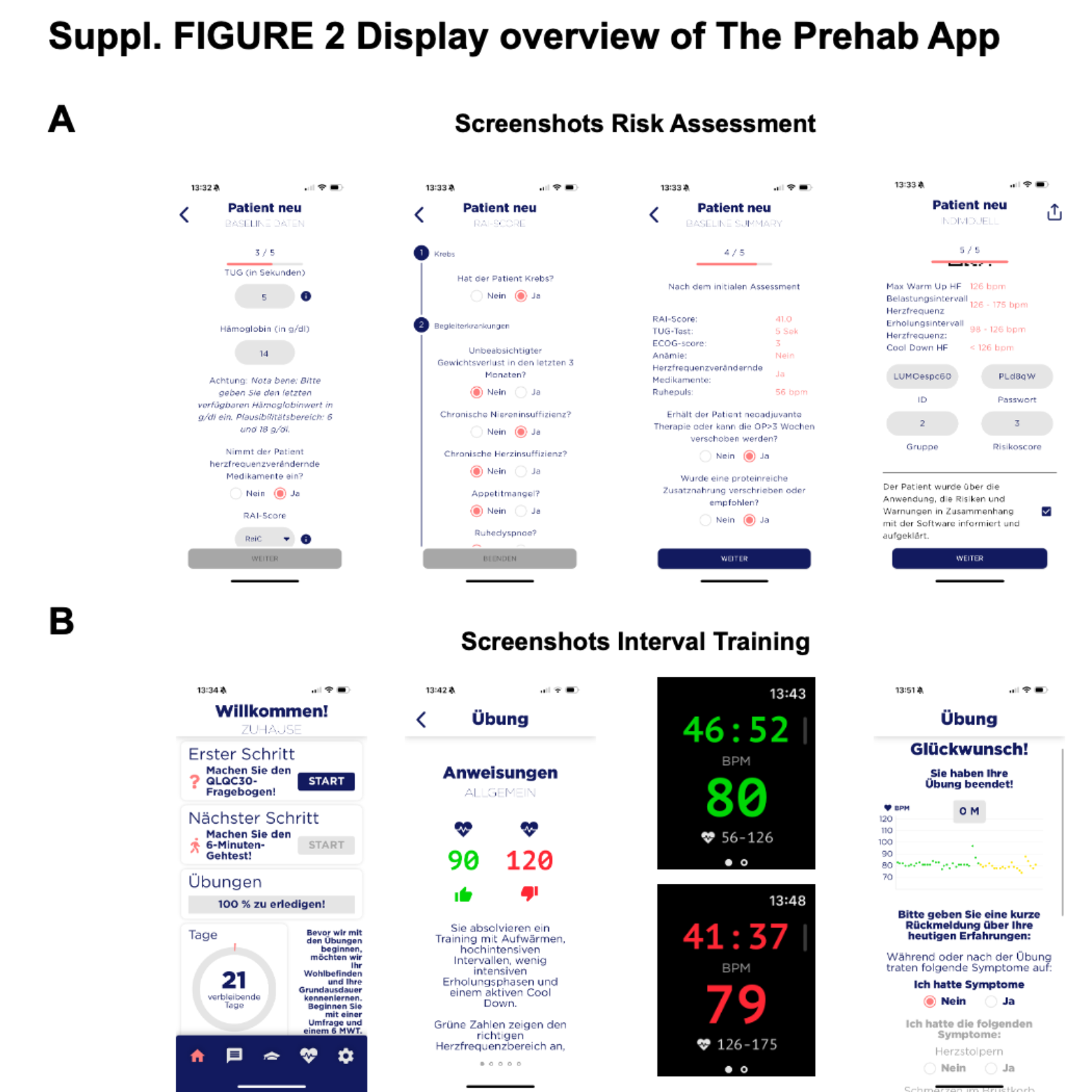

Supplement: Multimedia Appendix 2 [file mhealth_v13i1e55298_app2.png]

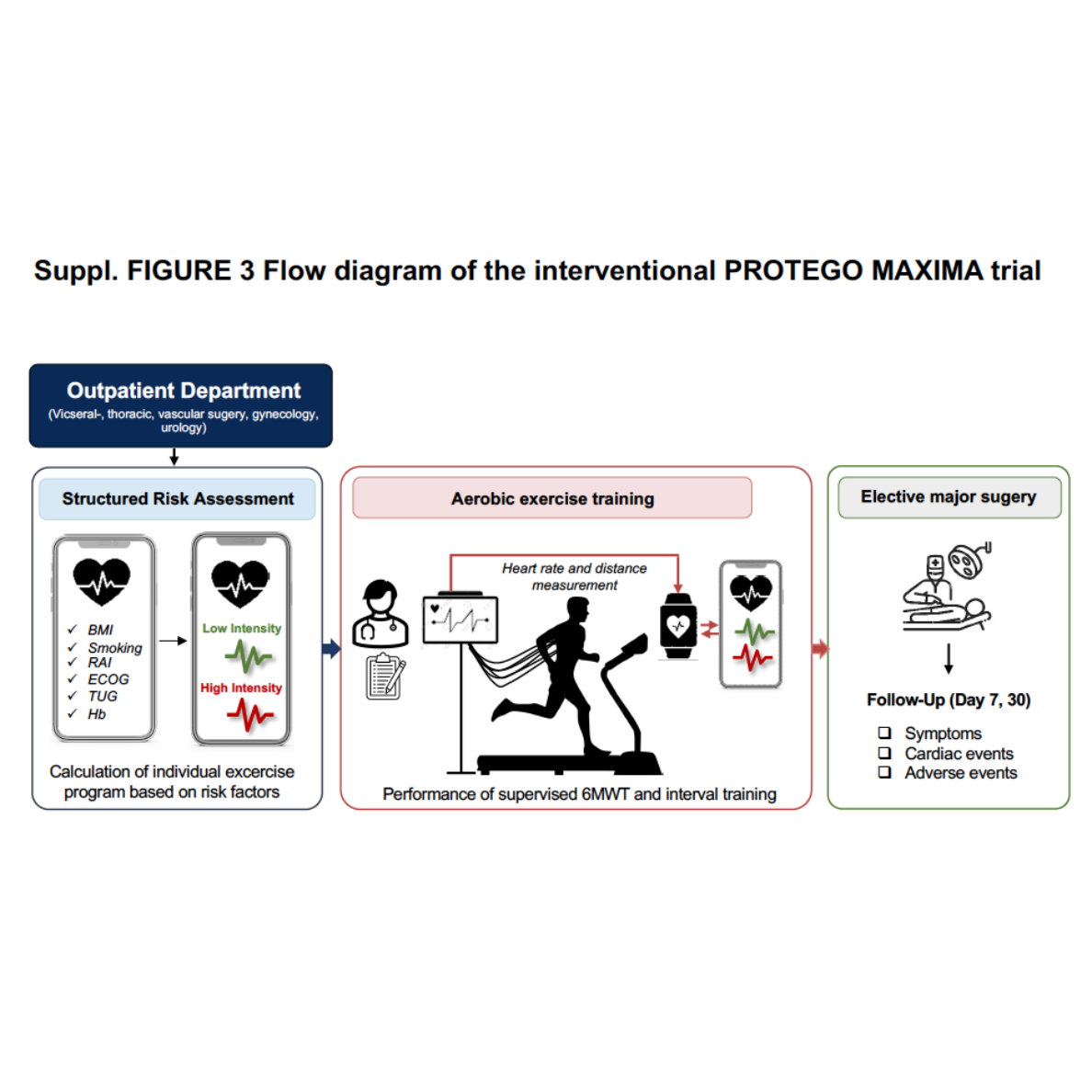

Supplement: Multimedia Appendix 3 [file mhealth_v13i1e55298_app3.png]

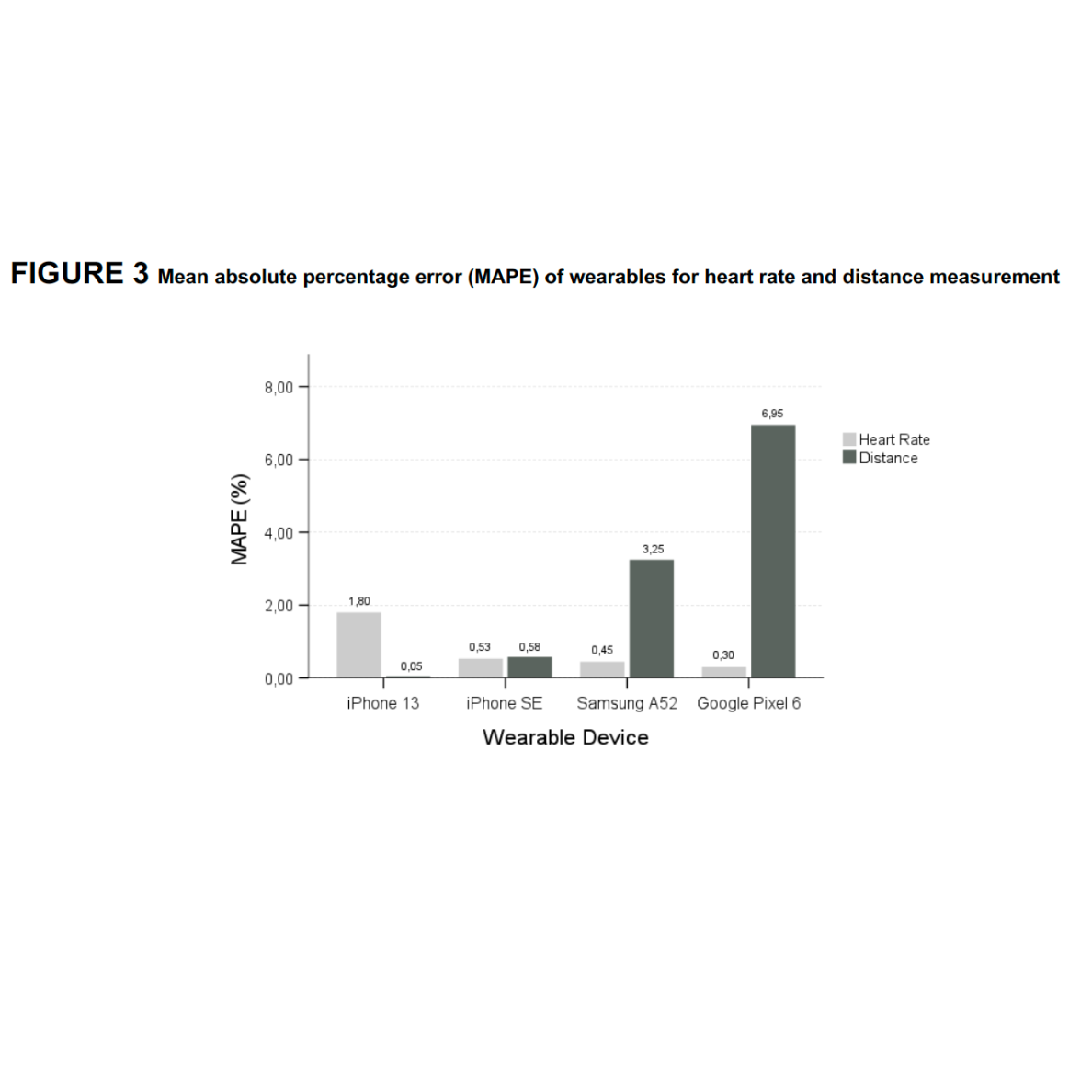

Supplement: Multimedia Appendix 6 [file mhealth_v13i1e55298_app6.png]
