# Supplementary material for: Validity, Accuracy, and Safety Assessment of an Aerobic Interval Training Using an App-Based Prehabilitation Program (PROTEGO MAXIMA Trial) Before Major Surgery: Prospective, Interventional Pilot Study
Source: JMIR Mhealth Uhealth. 2025 Feb 10;13:e55298. doi: 10.2196/55298 (PMC11851035; doi:10.2196/55298)
Supplement: Multimedia Appendix 5 [file mhealth_v13i1e55298_app5.docx]

| **Variables** | **Healthy students**  **(n = 22)** | **Patients**  **(n = 128)** |  | ***P* Value** |  |
| --- | --- | --- | --- | --- | --- |
| **iPhone 13 + App Watch 7**  Mean difference, bpm  MAPE (%) | 0.77  -0.33 | 3.02  -2.13 |  | .611  .559 |  |
| **iPhone SE + App Watch 13**  Mean difference, bpm  MAPE (%) | -0.42  0.59 | 1.55  -1.06 |  | .237  .268 |  |
| **Samsung A52 + Sam Watch 4**  Mean difference, bpm  MAPE (%) | 0.24  1.18 | 2.48  -1.27 |  | .728  .521 |  |
| **Google Pixel 6 + Sam Watch 4**  Mean difference, bpm  MAPE (%) | 1.30  0.19 | 1.63  -0.41 |  | .690  .510 |  |
| The values given are mean (standard deviation) unless indicated otherwise·  MAPE: mean absolute percentage; LoA: 95% limits of agreement; bpm: beats per minute; SD: standard  deviation· | | | | | |
