# Supplementary material for: Validity, Accuracy, and Safety Assessment of an Aerobic Interval Training Using an App-Based Prehabilitation Program (PROTEGO MAXIMA Trial) Before Major Surgery: Prospective, Interventional Pilot Study
Source: JMIR Mhealth Uhealth. 2025 Feb 10;13:e55298. doi: 10.2196/55298 (PMC11851035; doi:10.2196/55298)
Supplement: Multimedia Appendix 4 [file mhealth_v13i1e55298_app4.pdf]

CONSORT-EHEALTH (V 1.6.1) - Submission/Publication Form

The CONSORT-EHEALTH checklist is intended for authors of randomized trials evaluating web-based and Internet-based applications/interventions, including mobile interventions, electronic games (incl multiplayer games), social media, certain telehealth applications, and other interactive and/or networked electronic applications. Some of the items (e.g. all subitems under item 5 - description of the intervention) may also be applicable for other study designs.

The goal of the CONSORT EHEALTH checklist and guideline is to be
a) a guide for reporting for authors of RCTs,
b) to form a basis for appraisal of an ehealth trial (in terms of validity)

CONSORT-EHEALTH items/subitems are MANDATORY reporting items for studies published in the Journal of Medical Internet Research and other journals / scientific societies endorsing the checklist.

Items numbered 1., 2., 3., 4a., 4b etc are original CONSORT or CONSORT-NPT (non-pharmacologic treatment) items.
Items with Roman numerals (i., ii, iii, iv etc.) are CONSORT-EHEALTH extensions/clarifications.

As the CONSORT-EHEALTH checklist is still considered in a formative stage, we would ask that you also RATE ON A SCALE OF 1-5 how important/useful you feel each item is FOR THE PURPOSE OF THE CHECKLIST and reporting guideline (optional).

Mandatory reporting items are marked with a red *.
In the textboxes, either copy & paste the relevant sections from your manuscript into this form - please include any quotes from your manuscript in QUOTATION MARKS,
or answer directly by providing additional information not in the manuscript, or elaborating on why the item was not relevant for this study.

YOUR ANSWERS WILL BE PUBLISHED AS A SUPPLEMENTARY FILE TO YOUR PUBLICATION IN JMIR AND ARE CONSIDERED PART OF YOUR PUBLICATION (IF ACCEPTED).
Please fill in these questions diligently. Information will not be copyedited, so please use proper spelling and grammar, use correct capitalization, and avoid abbreviations.

DO NOT FORGET TO SAVE AS PDF _AND_ CLICK THE SUBMIT BUTTON SO YOUR ANSWERS ARE IN OUR DATABASE !!!

Citation Suggestion (if you append the pdf as Appendix we suggest to cite this paper in the caption):
Eysenbach G, CONSORT-EHEALTH Group
CONSORT-EHEALTH: Improving and Standardizing Evaluation Reports of Web-based and Mobile Health Interventions
J Med Internet Res 2011;13(4):e126
URL: http://www.jmir.org/2011/4/e126/
doi: 10.2196/jmir.1923
PMID: 22209829

sarafatima189@gmail.com Konto wechseln
Nicht freigegeben
Entwurf gespeichert

* Gibt eine erforderliche Frage an

Your name *
First Last

Fatima

Primary Affiliation (short), City, Country *
University of Toronto, Toronto, Canada

University Frankfurt am Main, Frankfurt, Germa

Your e-mail address *
abc@gmail.com

sarafatima189@gmail.com

Title of your manuscript *
Provide the (draft) title of your manuscript.

Validity, accuracy, and safety assessment of an aerobic interval training using an App-based prehabilitation program before major surgery: PROTEGO-MAXIMA-Trial

Name of your App/Software/Intervention *
If there is a short and a long/alternate name, write the short name first and add the long name in brackets.

PROTEGO MAXIMA

Evaluated Version (if any)
e.g. "V1", "Release 2017-03-01", "Version 2.0.27913"

Version 1

Language(s) *
What language is the intervention/app in? If multiple languages are available, separate by comma (e.g. "English, French")

German, English

URL of your Intervention Website or App
e.g. a direct link to the mobile app on app in appstore (itunes, Google Play), or URL of the website. If the intervention is a DVD or hardware, you can also link to an Amazon page.

www.capreolos.com

URL of an image/screenshot (optional)

N.A.

Accessibility *
Can an enduser access the intervention presently?

- ( ) access is free and open
- (x) access only for special usergroups, not open
- ( ) access is open to everyone, but requires payment/subscription/in-app purchases
- ( ) app/intervention no longer accessible
- ( ) Sonstiges:

Primary Medical Indication/Disease/Condition *
e.g. "Stress", "Diabetes", or define the target group in brackets after the condition, e.g. "Autism (Parents of children with)", "Alzheimers (Informal Caregivers of)"

Major surgery

Primary Outcomes measured in trial *
comma-separated list of primary outcomes reported in the trial

Validity of the heart rate measurement of smartwatches in comparison to a certified ECG

Secondary/other outcomes
Are there any other outcomes the intervention is expected to affect?

safety, complications rates associated with the use during a moderate to strenous exercise

Recommended "Dose" *
What do the instructions for users say on how often the app should be used?

- (x) Approximately Daily
- ( ) Approximately Weekly
- ( ) Approximately Monthly
- ( ) Approximately Yearly
- ( ) "as needed"
- ( ) Sonstiges:

Approx. Percentage of Users (starters) still using the app as recommended after 3 months *

- (x) unknown / not evaluated
- ( ) 0-10%
- ( ) 11-20%
- ( ) 21-30%
- ( ) 31-40%
- ( ) 41-50%
- ( ) 51-60%
- ( ) 61-70%
- ( ) 71%-80%
- ( ) 81-90%
- ( ) 91-100%
- ( ) Sonstiges:

Overall, was the app/intervention effective? *

- (x) yes: all primary outcomes were significantly better in intervention group vs control
- ( ) partly: SOME primary outcomes were significantly better in intervention group vs control
- ( ) no statistically significant difference between control and intervention
- ( ) potentially harmful: control was significantly better than intervention in one or more outcomes
- ( ) inconclusive: more research is needed
- ( ) Sonstiges:

Article Preparation Status/Stage *
At which stage in your article preparation are you currently (at the time you fill in this form)

- ( ) not submitted yet - in early draft status
- ( ) not submitted yet - in late draft status, just before submission
- ( ) submitted to a journal but not reviewed yet
- (x) submitted to a journal and after receiving initial reviewer comments
- ( ) submitted to a journal and accepted, but not published yet
- ( ) published
- ( ) Sonstiges:

Journal *
If you already know where you will submit this paper (or if it is already submitted), please provide the journal name (if it is not JMIR, provide the journal name under "other")

- ( ) not submitted yet / unclear where I will submit this
- (x) Journal of Medical Internet Research (JMIR)
- ( ) JMIR mHealth and UHealth
- ( ) JMIR Serious Games
- ( ) JMIR Mental Health
- ( ) JMIR Public Health
- ( ) JMIR Formative Research
- ( ) Other JMIR sister journal
- ( ) Sonstiges:

Is this a full powered effectiveness trial or a pilot/feasibility trial? *

- (x) Pilot/feasibility
- ( ) Fully powered

Manuscript tracking number *
If this is a JMIR submission, please provide the manuscript tracking number under "other" (The ms tracking number can be found in the submission acknowledgement email, or when you login as author in JMIR. If the paper is already published in JMIR, then the ms tracking number is the four-digit number at the end of the DOI, to be found at the bottom of each published article in JMIR)

- ( ) no ms number (yet) / not (yet) submitted to / published in JMIR
- (x) Sonstiges: ms#59513

TITLE AND ABSTRACT

1a) TITLE: Identification as a randomized trial in the title

1a) Does your paper address CONSORT item 1a? *
I.e does the title contain the phrase "Randomized Controlled Trial"? (if not, explain the reason under "other")

- ( ) yes
- (x) Sonstiges: No

1a-i) Identify the mode of delivery in the title
Identify the mode of delivery. Preferably use "web-based" and/or "mobile" and/or "electronic game" in the title. Avoid ambiguous terms like "online", "virtual", "interactive". Use "Internet-based" only if Intervention includes non-web-based Internet components (e.g. email), use "computer-based" or "electronic" only if offline products are used. Use "virtual" only in the context of "virtual reality" (3-D worlds). Use "online" only in the context of "online support groups". Complement or substitute product names with broader terms for the class of products (such as "mobile" or "smart phone" instead of "iphone"), especially if the application runs on different platforms.

subitem not at all important 1 ( ) 2 ( ) 3 (x) 4 ( ) 5 ( ) essential
Auswahl löschen

Does your paper address subitem 1a-i? *
Copy and paste relevant sections from manuscript title (include quotes in quotation marks "like this" to indicate direct quotes from your manuscript), or elaborate on this item by providing additional information not in the ms, or briefly explain why the item is not applicable/relevant for your study

"App-based prehabilitation program "

1a-ii) Non-web-based components or important co-interventions in title
Mention non-web-based components or important co-interventions in title, if any (e.g., "with telephone support").

subitem not at all important 1 ( ) 2 ( ) 3 (x) 4 ( ) 5 ( ) essential
Auswahl löschen

Does your paper address subitem 1a-ii?
Copy and paste relevant sections from manuscript title (include quotes in quotation marks "like this" to indicate direct quotes from your manuscript), or elaborate on this item by providing additional information not in the ms, or briefly explain why the item is not applicable/relevant for your study

"App-based prehabilitation program "

1a-iii) Primary condition or target group in the title
Mention primary condition or target group in the title, if any (e.g., "for children with Type I Diabetes") Example: A Web-based and Mobile Intervention with Telephone Support for Children with Type I Diabetes: Randomized Controlled Trial

subitem not at all important 1 ( ) 2 ( ) 3 (x) 4 ( ) 5 ( ) essential
Auswahl löschen

Does your paper address subitem 1a-iii? *
Copy and paste relevant sections from manuscript title (include quotes in quotation marks "like this" to indicate direct quotes from your manuscript), or elaborate on this item by providing additional information not in the ms, or briefly explain why the item is not applicable/relevant for your study

"Validity, accuracy, and safety assessment of an aerobic interval training using an App-based prehabilitation program before major surgery"

1b) ABSTRACT: Structured summary of trial design, methods, results, and conclusions
NPT extension: Description of experimental treatment, comparator, care providers, centers, and blinding status.

1b-i) Key features/functionalities/components of the intervention and comparator in the METHODS section of the ABSTRACT
Mention key features/functionalities/components of the intervention and comparator in the abstract. If possible, also mention theories and principles used for designing the site. Keep in mind the needs of systematic reviewers and indexers by including important synonyms. (Note: Only report in the abstract what the main paper is reporting. If this information is missing from the main body of text, consider adding it)

subitem not at all important 1 ( ) 2 ( ) 3 (x) 4 ( ) 5 ( ) essential
Auswahl löschen

Does your paper address subitem 1b-i? *
Copy and paste relevant sections from the manuscript abstract (include quotes in quotation marks "like this" to indicate direct quotes from your manuscript), or elaborate on this item by providing additional information not in the ms, or briefly explain why the item is not applicable/relevant for your study

Background: Major surgery is associated with substantial morbidity and reduced quality of life, particularly in the elderly and frail with impaired functional capacity. Multimodal prehabilitation can enhance functional recovery after surgery and reduce postoperative complications. Digital prehabilitation applications gained momentum being a resource-sparing tool improving patients' preoperative status. Still, little is known regarding their safety and accuracy as medical devices.
Objectives: to test the accuracy and validity of a new software in comparison to gold standard electrocardiogram-based heart rate measurement.
Methods: The PROTEGO MAXIMA trial was a prospective interventional pilot trial assessing the validity, accuracy, and safety of an app-based exercise program. The Prehab App calculates an individual, risk-stratified aerobic interval training based on risk factors and uses wearables for heart rate monitoring. Healthy students and patients undergoing major surgery were enrolled. Structured risk assessment was performed followed by a 6-min walking test and a 37-min supervised interval. During the exercise, patients wore App-linked wearables for heart rate and distance measurement, which were compared to standard ECG (electrocardiography) and treadmill measurements. Safety, accuracy, and usability assessments included testing of alarm signals, and occurrence of adverse events were primary and secondary outcome measures
Results: Seventy-five participants were included. Mean heart rate differences between wearables and standard ECG were ≤5bpm (beats per minute) with a mean absolute percentage error (MAPE) ≤5%. Regression analysis revealed a significant impact of the body mass index (BMI) (OR (odds ratio) 0·90, 95% CI (confidence interval) [0·82, 0·98], P = ·02) and TUG (timed up and go test)-score (OR 0·12, 95% CI [0·03, 0·55], P = ·006) on the accuracy of heart rate measurement. Twenty-nine (39%) patients experienced adverse events: pain (42%), ECG electrode-related skin irritations (17%), dizziness (17%), shortness of breath (17%), and fatigue (8%). No cardiovascular, or serious adverse event was reported, and no serious device deficiency was detected. There were no signals from lab values taken before and after the 6MWT and the exercise that indicated clinical meaningful overexertion: lactate (mmol/l) differences in mean plus range: 0.04 (-3 to 6); P = .47; Creatinine Kinase (U/L): 12 (-7 to 43); P < .001; Sodium (mmol/L: -2 (-11 to 12); P < .001.
Conclusions: The interventional trial showed high safety of the exercise program and accuracy of heart rate measurements using commercial wearables in patients before major surgery allowing a remote setting in the future.
Funding: Else-Kroener-Fresenius Stiftung (number: 2021_EKTP10)

1b-ii) Level of human involvement in the METHODS section of the ABSTRACT
Clarify the level of human involvement in the abstract, e.g., use phrases like "fully automated" vs. "therapist/nurse/care provider/physician-assisted" (mention number and expertise of providers involved, if any). (Note: Only report in the abstract what the main paper is reporting. If this information is missing from the main body of text, consider adding it)

subitem not at all important 1 ( ) 2 (x) 3 ( ) 4 ( ) 5 ( ) essential
Auswahl löschen

Does your paper address subitem 1b-ii?
Copy and paste relevant sections from the manuscript abstract (include quotes in quotation marks "like this" to indicate direct quotes from your manuscript), or elaborate on this item by providing additional information not in the ms, or briefly explain why the item is not applicable/relevant for your study

"Healthy students and patients undergoing major surgery were enrolled. Structured risk assessment was performed followed by a 6-min walking test and a 37-min supervised interval. "

1b-iii) Open vs. closed, web-based (self-assessment) vs. face-to-face assessments in the METHODS section of the ABSTRACT
Mention how participants were recruited (online vs. offline), e.g., from an open access website or from a clinic or a closed online user group (closed usergroup trial), and clarify if this was a purely web-based trial, or there were face-to-face components (as part of the intervention or for assessment). Clearly say if outcomes were self-assessed through questionnaires (as common in web-based trials). Note: In traditional offline trials, an open trial (open-label trial) is a type of clinical trial in which both the researchers and participants know which treatment is being administered. To avoid confusion, use "blinded" or "unblinded" to indicated the level of blinding instead of "open", as "open" in web-based trials usually refers to "open access" (i.e. participants can self-enrol). (Note: Only report in the abstract what the main paper is reporting. If this information is missing from the main body of text, consider adding it)

subitem not at all important 1 ( ) 2 (x) 3 ( ) 4 ( ) 5 ( ) essential
Auswahl löschen

Does your paper address subitem 1b-iii?
Copy and paste relevant sections from the manuscript abstract (include quotes in quotation marks "like this" to indicate direct quotes from your manuscript), or elaborate on this item by providing additional information not in the ms, or briefly explain why the item is not applicable/relevant for your study

"In brief, first 10 healthy students, recruited at the Sports Institute at the Goethe University Frankfurt to get a baseline dataset from healthy volunteers.In the next step 65 patients aged 18 years and older, able to understand and perform the endurance exercise program, and scheduled for an elective major surgery before major surgeries were recruited at the University Hospital Frankfurt"

1b-iv) RESULTS section in abstract must contain use data
Report number of participants enrolled/assessed in each group, the use/uptake of the intervention (e.g., attrition/adherence metrics, use over time, number of logins etc.), in addition to primary/secondary outcomes. (Note: Only report in the abstract what the main paper is reporting. If this information is missing from the main body of text, consider adding it)

subitem not at all important 1 ( ) 2 ( ) 3 ( ) 4 (x) 5 ( ) essential
Auswahl löschen

Does your paper address subitem 1b-iv?
Copy and paste relevant sections from the manuscript abstract (include quotes in quotation marks "like this" to indicate direct quotes from your manuscript), or elaborate on this item by providing additional information not in the ms, or briefly explain why the item is not applicable/relevant for your study

"Seventy-five participants were included"

1b-v) CONCLUSIONS/DISCUSSION in abstract for negative trials
Conclusions/Discussions in abstract for negative trials: Discuss the primary outcome - if the trial is negative (primary outcome not changed), and the intervention was not used, discuss whether negative results are attributable to lack of uptake and discuss reasons. (Note: Only report in the abstract what the main paper is reporting. If this information is missing from the main body of text, consider adding it)

subitem not at all important 1 ( ) 2 ( ) 3 ( ) 4 (x) 5 ( ) essential
Auswahl löschen

Does your paper address subitem 1b-v?
Copy and paste relevant sections from the manuscript abstract (include quotes in quotation marks "like this" to indicate direct quotes from your manuscript), or elaborate on this item by providing additional information not in the ms, or briefly explain why the item is not applicable/relevant for your study

"The interventional trial showed high safety of the exercise program and accuracy of heart rate measurements using commercial wearables in patients before major surgery allowing a remote setting in the future"

INTRODUCTION

2a) In INTRODUCTION: Scientific background and explanation of rationale

2a-i) Problem and the type of system/solution
Describe the problem and the type of system/solution that is object of the study: intended as stand-alone intervention vs. incorporated in broader health care program? Intended for a particular patient population? Goals of the intervention, e.g., being more cost-effective to other interventions, replace or complement other solutions? (Note: Details about the intervention are provided in "Methods" under 5)

subitem not at all important 1 ( ) 2 ( ) 3 ( ) 4 (x) 5 ( ) essential
Auswahl löschen

Does your paper address subitem 2a-i? *
Copy and paste relevant sections from the manuscript (include quotes in quotation marks "like this" to indicate direct quotes from your manuscript), or elaborate on this item by providing additional information not in the ms, or briefly explain why the item is not applicable/relevant for your study

"Postoperative complications are experienced by 15-40% of patients undergoing major surgery, potentially leading to life-threatening complications, decline of quality of life or reduced physical functioning. Adverse events related to surgical procedures account for tremendous financial burden through additional costs caused by intensive care treatment, reoperations, or prolonged hospitalization "

2a-ii) Scientific background, rationale: What is known about the (type of) system
Scientific background, rationale: What is known about the (type of) system that is the object of the study (be sure to discuss the use of similar systems for other conditions/diagnoses, if appropiate), motivation for the study, i.e. what are the reasons for and what is the context for this specific study, from which stakeholder viewpoint is the study performed, potential impact of findings [2]. Briefly justify the choice of the comparator.

subitem not at all important 1 ( ) 2 ( ) 3 ( ) 4 (x) 5 ( ) essential
Auswahl löschen

Does your paper address subitem 2a-ii? *
Copy and paste relevant sections from the manuscript (include quotes in quotation marks "like this" to indicate direct quotes from your manuscript), or elaborate on this item by providing additional information not in the ms, or briefly explain why the item is not applicable/relevant for your study

"Importantly, the physical ability to recuperate from physiological stress induced by surgery and an aggressive postoperative metabolism, highly correlates with the preoperative health status and physical functioning. Therefore, preoperative exercise interventions aim at improving patients' aerobic and muscle strength capacity to significantly facilitate postoperative recovery. Prehabilitation as an emerging field in the perioperative care addresses modifiable risk factors before surgery comprising exercise training, nutritional support, and psycho-cognitive training."

2b) In INTRODUCTION: Specific objectives or hypotheses

Does your paper address CONSORT subitem 2b? *
Copy and paste relevant sections from the manuscript (include quotes in quotation marks "like this" to indicate direct quotes from your manuscript), or elaborate on this item by providing additional information not in the ms, or briefly explain why the item is not applicable/relevant for your study

"The hypothesis was, that the software will achieve similar accuracy and validity in heart-rate measurement during a 6-minute walking test and a full 47-minute interval training as a [illegible]

certified electrocardiogram which is regarded to be the gold standard.

METHODS

3a) Description of trial design (such as parallel, factorial) including allocation ratio

Does your paper address CONSORT subitem 3a? *
Copy and paste relevant sections from the manuscript (include quotes in quotation marks "like this" to indicate direct quotes from your manuscript), or elaborate on this item by providing additional information not in the ms, or briefly explain why the item is not applicable/relevant for your study

"The prospective interventional PROTEGO MAXIMA trial was designed to evaluate validity, accuracy, and safety of a prehabilitation app. The reporting has been carried out in accordance with the STARDreporting guidelines. It included healthy students and patients before elective major surgery between March 25, 2022, and September 29, 2022, at the University Hospital Frankfurt. The study protocol has been previously published."

3b) Important changes to methods after trial commencement (such as eligibility criteria), with reasons

Does your paper address CONSORT subitem 3b? *
Copy and paste relevant sections from the manuscript (include quotes in quotation marks "like this" to indicate direct quotes from your manuscript), or elaborate on this item by providing additional information not in the ms, or briefly explain why the item is not applicable/relevant for your study

No changing of methods planned. If unexpected events occurred, the intervention was stopped.

3b-i) Bug fixes, Downtimes, Content Changes
Bug fixes, Downtimes, Content Changes: ehealth systems are often dynamic systems. A description of changes to methods therefore also includes important changes made on the intervention or comparator during the trial (e.g., major bug fixes or changes in the functionality or content) (5-iii) and other "unexpected events" that may have influenced study design such as staff changes, system failures/downtimes, etc. [2].

	1	2	3	4	5	
subitem not at all important	○	◉	○	○	○	essential

Auswahl löschen

Does your paper address subitem 3b-i?
Copy and paste relevant sections from the manuscript (include quotes in quotation marks "like this" to indicate direct quotes from your manuscript), or elaborate on this item by providing additional information not in the ms, or briefly explain why the item is not applicable/relevant for your study

No changing of methods planned. If unexpected events occurred, the intervention was stopped.

4a) Eligibility criteria for participants

Does your paper address CONSORT subitem 4a? *
Copy and paste relevant sections from the manuscript (include quotes in quotation marks "like this" to indicate direct quotes from your manuscript), or elaborate on this item by providing additional information not in the ms, or briefly explain why the item is not applicable/relevant for your study

"In brief, first 10 healthy students, recruited at the Sports Institute at the Goethe University Frankfurt to get a baseline dataset from healthy volunteers.In the next step 65 patients aged 18 years and older, able to understand and perform the endurance exercise program, and scheduled for an elective major surgery before major surgeries were recruited at the University Hospital Frankfurt. Gastrointestinal resection, resection of the hepatobiliary pancreatic system, endocrine glands, lung or bronchus, splenectomy, abdominal wall hernia, urological or gynecologic resections, or vascular surgery without cardiovascular procedures were eligible for the trial. "

4a-i) Computer / Internet literacy
Computer / Internet literacy is often an implicit "de facto" eligibility criterion - this should be explicitly clarified.

	1	2	3	4	5	
subitem not at all important	○	◉	○	○	○	essential

Auswahl löschen

Does your paper address subitem 4a-i?
Copy and paste relevant sections from the manuscript (include quotes in quotation marks "like this" to indicate direct quotes from your manuscript), or elaborate on this item by providing additional information not in the ms, or briefly explain why the item is not applicable/relevant for your study

No computer/Internet literacy used

4a-ii) Open vs. closed, web-based vs. face-to-face assessments:
Open vs. closed, web-based vs. face-to-face assessments: Mention how participants were recruited (online vs. offline), e.g., from an open access website or from a clinic, and clarify if this was a purely web-based trial, or there were face-to-face components (as part of the intervention or for assessment), i.e., to what degree got the study team to know the participant. In online-only trials, clarify if participants were quasi-anonymous and whether having multiple identities was possible or whether technical or logistical measures (e.g., cookies, email confirmation, phone calls) were used to detect/prevent these.

	1	2	3	4	5	
subitem not at all important	○	○	◉	○	○	essential

Auswahl löschen

Does your paper address subitem 4a-ii? *
Copy and paste relevant sections from the manuscript (include quotes in quotation marks "like this" to indicate direct quotes from your manuscript), or elaborate on this item by providing additional information not in the ms, or briefly explain why the item is not applicable/relevant for your study

Participants were recruited from the clinic (face-to-face assessment)

4a-iii) Information giving during recruitment
Information given during recruitment. Specify how participants were briefed for recruitment and in the informed consent procedures (e.g., publish the informed consent documentation as appendix, see also item X26), as this information may have an effect on user self-selection, user expectation and may also bias results.

	1	2	3	4	5	
subitem not at all important	○	○	◉	○	○	essential

Auswahl löschen

Does your paper address subitem 4a-iii?
Copy and paste relevant sections from the manuscript (include quotes in quotation marks "like this" to indicate direct quotes from your manuscript), or elaborate on this item by providing additional information not in the ms, or briefly explain why the item is not applicable/relevant for your study

"All participants provided written informed consent. "

4b) Settings and locations where the data were collected

Does your paper address CONSORT subitem 4b? *
Copy and paste relevant sections from the manuscript (include quotes in quotation marks "like this" to indicate direct quotes from your manuscript), or elaborate on this item by providing additional information not in the ms, or briefly explain why the item is not applicable/relevant for your study

"As this is a first in human use pilot trial, we broadened the scope of data collection to as many as possible scenarios reaching from healthy students with anticipated textbook outcomes as well as normal and high-risk patients. Data of all patients were stored in the electronic case report form (eCRF) using the SecuTrial System, a web-based data management application complying with all required regulations. To assess the quality of data and auditing trial conduct, independent reviews were performed by monitors following the monitoring plan of the clinical trial. "

4b-i) Report if outcomes were (self-)assessed through online questionnaires
Clearly report if outcomes were (self-)assessed through online questionnaires (as common in web-based trials) or otherwise.

	1	2	3	4	5	
subitem not at all important	○	○	◉	○	○	essential

Auswahl löschen

Does your paper address subitem 4b-i? *
Copy and paste relevant sections from the manuscript (include quotes in quotation marks "like this" to indicate direct quotes from your manuscript), or elaborate on this item by providing additional information not in the ms, or briefly explain why the item is not applicable/relevant for your study

All data were assessed by the study doctors.

4b-ii) Report how institutional affiliations are displayed
Report how institutional affiliations are displayed to potential participants [on ehealth media], as affiliations with prestigious hospitals or universities may affect volunteer rates, use, and reactions with regards to an intervention.(Not a required item – describe only if this may bias results)

	1	2	3	4	5	
subitem not at all important	○	◉	○	○	○	essential

Auswahl löschen

Does your paper address subitem 4b-ii?
Copy and paste relevant sections from the manuscript (include quotes in quotation marks "like this" to indicate direct quotes from your manuscript), or elaborate on this item by providing additional information not in the ms, or briefly explain why the item is not applicable/relevant for your study

Item is not required and may not bias results.

5) The interventions for each group with sufficient details to allow replication, including how and when they were actually administered

5-i) Mention names, credential, affiliations of the developers, sponsors, and owners
Mention names, credential, affiliations of the developers, sponsors, and owners [6] (if authors/evaluators are owners or developer of the software, this needs to be declared in a "Conflict of interest" section or mentioned elsewhere in the manuscript).

	1	2	3	4	5	
subitem not at all important	○	○	◉	○	○	essential

Auswahl löschen

Does your paper address subitem 5-i?
Copy and paste relevant sections from the manuscript (include quotes in quotation marks "like this" to indicate direct quotes from your manuscript), or elaborate on this item by providing additional information not in the ms, or briefly explain why the item is not applicable/relevant for your study

"The Prehab App is developed by the Capreolos GmbH, a spin-off company of the Goethe University as medical device class IIa according to the MDR regulations. Currently, it is not yet a CE-certified medical device."

5-ii) Describe the history/development process
Describe the history/development process of the application and previous formative evaluations (e.g., focus groups, usability testing), as these will have an impact on adoption/use rates and help with interpreting results.

	1	2	3	4	5	
subitem not at all important	○	○	○	◉	○	essential

Auswahl löschen

Does your paper address subitem 5-ii?
Copy and paste relevant sections from the manuscript (include quotes in quotation marks "like this" to indicate direct quotes from your manuscript), or elaborate on this item by providing additional information not in the ms, or briefly explain why the item is not applicable/relevant for your study

"The App is linked to a wrist-worn wearable (iOS (internet operating system) and Google wear OS based hardware) through a software application and provides an individualized, digitized prehabilitation program for patients undergoing major surgery to improve their functional capacity using aerobic endurance exercise training. Based on the patient's risk factors (ECOG Score, RAI Score, TUG, hemoglobin values, and the presence of heart rate changing medication), and the resting heart rate, an individual interval training with a target heart rate range for a moderate to vigorous training intensity (50 to 80% of the maximum heart rate) is calculated using the Karvonen method [24]. The predefined exercising program is then automatically uploaded to the patient's smartphone and is ready to use with a provided wearable. By measuring heart rate and distance during the exercise, the App provides feedback to the patients on their physical performance and potential alarms, if the measured heart rate exceeds or falls below the suggested ranges (automated safety audit). Further, the App of the treating physician will be linked to the patients' App, enabling supervision by receiving the same alarms as defined in the safety audit and derived from the DIN ISO 14971 compliant risk management. Having performed the individual exercises a patient reported outcome form will collect symptoms during the exercise that leads to feeds in the safety audit in case of more than two reported symptoms. In the final intended use, the patients will perform the training -three to four times a week, selecting the type of training (i.e., cycling, walking, running) during any time of the day, facilitating a home-based patient-empowering environment. A 6-min walking test (6MWT) at the beginning and end of the training period measured the baseline and at the end of the program. The Quality-of-Life Questionnaire for cancer patients containing 30 questions (QLQC30) will assess quality of life before and at the end of the training period. "

5-iii) Revisions and updating
Revisions and updating. Clearly mention the date and/or version number of the application/intervention (and comparator, if applicable) evaluated, or describe whether the intervention underwent major changes during the evaluation process, or whether the development and/or content was "frozen" during the trial. Describe dynamic components such as news feeds or changing content which may have an impact on the replicability of the intervention (for unexpected events see item 3b).

	1	2	3	4	5	
subitem not at all important	○	○	○	◉	○	essential

Auswahl löschen

Does your paper address subitem 5-iii?
Copy and paste relevant sections from the manuscript (include quotes in quotation marks "like this" to indicate direct quotes from your manuscript), or elaborate on this item by providing additional information not in the ms, or briefly explain why the item is not applicable/relevant for your study

This is a pilot study to assess the validity, accuracy and safety. During the trial, the software didn't underwent any changes.

5-iv) Quality assurance methods
Provide information on quality assurance methods to ensure accuracy and quality of information provided [1], if applicable.

	1	2	3	4	5	
subitem not at all important	○	○	◉	○	○	essential

Auswahl löschen

Does your paper address subitem 5-iv?
Copy and paste relevant sections from the manuscript (include quotes in quotation marks "like this" to indicate direct quotes from your manuscript), or elaborate on this item by providing additional information not in the ms, or briefly explain why the item is not applicable/relevant for your study

"The aerobic exercise training was continuously supervised by a physician to ensure a safe environment. Secondary safety measures were based on blood samples -reported elsewhere- that were taken before and 15 minutes after the interval training (including creatinephosphatekinase, creatine-kinase-MB, lactate dehydrogenase, glucose, sodium, potassium, chloride, c-reactive protein, blood carbondioxide, lactate, and cell-free DNA (desoxy ribonucleic acid)). Moreover, awareness of participants for specific alarm signals displayed on the wearable devices, either by color or by haptic vibration when exceeding or falling below the defined heart rate ranges were assessed. Symptoms, cardiac events, adverse events (AE), serious adverse events (SAE) were assessed during the exercise and through structured telephone interviews or by visiting patients in the hospital (in case they already underwent their planned surgery) at day 7 and 30 days after the exercise intervention. "

5-v) Ensure replicability by publishing the source code, and/or providing screenshots/screen-capture video, and/or providing flowcharts of the algorithms used
Ensure replicability by publishing the source code, and/or providing screenshots/screen-capture video, and/or providing flowcharts of the algorithms used. Replicability (i.e., other researchers should in principle be able to replicate the study) is a hallmark of scientific reporting.

	1	2	3	4	5	
subitem not at all important	○	○	○	◉	○	essential

Auswahl löschen

Does your paper address subitem 5-v?
Copy and paste relevant sections from the manuscript (include quotes in quotation marks "like this" to indicate direct quotes from your manuscript), or elaborate on this item by providing additional information not in the ms, or briefly explain why the item is not applicable/relevant for your study

Screen shots of the App will be provided as supplementaries in the Multimedia Appendix.

5-vi) Digital preservation
Digital preservation: Provide the URL of the application, but as the intervention is likely to change or disappear over the course of the years; also make sure the intervention is archived (Internet Archive, webcitation.org, and/or publishing the source code or screenshots/videos alongside the article). As pages behind login screens cannot be archived, consider creating demo pages which are accessible without login.

	1	2	3	4	5	
subitem not at all important	○	○	◉	○	○	essential

Auswahl löschen

Does your paper address subitem 5-vi?
Copy and paste relevant sections from the manuscript (include quotes in quotation marks "like this" to indicate direct quotes from your manuscript), or elaborate on this item by providing additional information not in the ms, or briefly explain why the item is not applicable/relevant for your study

https://capreolos.com/#prehab
Screen shots of the App will be provided as supplementaries in the Multimedia Appendix.

5-vii) Access
Access: Describe how participants accessed the application, in what setting/context, if they had to pay (or were paid) or not, whether they had to be a member of specific group. If known, describe how participants obtained "access to the platform and Internet" [1]. To ensure access for editors/reviewers/readers, consider to provide a "backdoor" login account or demo mode for reviewers/readers to explore the application (also important for archiving purposes, see vi).

	1	2	3	4	5	
subitem not at all important	○	○	◉	○	○	essential

Auswahl löschen

Does your paper address subitem 5-vii? *
Copy and paste relevant sections from the manuscript (include quotes in quotation marks "like this" to indicate direct quotes from your manuscript), or elaborate on this item by providing additional information not in the ms, or briefly explain why the item is not applicable/relevant for your study

The App was downloaded by participants on their. own smartphones from Google Play or App Store and was free of charge. The downloading process was supervised and assisted by the study doctors.

5-viii) Mode of delivery, features/functionalities/components of the intervention and comparator, and the theoretical framework
Describe mode of delivery, features/functionalities/components of the intervention and comparator, and the theoretical framework [6] used to design them (instructional strategy [1], behaviour change techniques, persuasive features, etc., see e.g., [7, 8] for terminology). This includes an in-depth description of the content (including where it is coming from and who developed it) [1]," whether [and how] it is tailored to individual circumstances and allows users to track their progress and receive feedback" [6]. This also includes a description of communication delivery channels and – if computer-mediated communication is a component – whether communication was synchronous or asynchronous [6]. It also includes information on presentation strategies [1], including page design principles, average amount of text on pages, presence of hyperlinks to other resources, etc. [1].

	1	2	3	4	5	
subitem not at all important	○	○	○	◉	○	essential

Auswahl löschen

Does your paper address subitem 5-viii? *
Copy and paste relevant sections from the manuscript (include quotes in quotation marks "like this" to indicate direct quotes from your manuscript), or elaborate on this item by providing additional information not in the ms, or briefly explain why the item is not applicable/relevant for your study

"The App is linked to a wrist-worn wearable (iOS (internet operating system) and Google wear OS based hardware) through a software application and provides an individualized, digitized prehabilitation program for patients undergoing major surgery to improve their functional capacity using aerobic endurance exercise training. Based on the patient's risk factors (ECOG Score, RAI Score, TUG, hemoglobin values, and the presence of heart rate changing medication), and the resting heart rate, an individual interval training with a target heart rate range for a moderate to vigorous training intensity (50 to 80% of the maximum heart rate) is calculated using the Karvonen method. The predefined exercising program is then automatically uploaded to the patient's smartphone and is ready to use with a provided wearable. By measuring heart rate and distance during the exercise, the App provides feedback to the patients on their physical performance and potential alarms, if the measured heart rate exceeds or falls below the suggested ranges (automated safety audit). Further, the App of the treating physician will be linked to the patients' App, enabling supervision by receiving the same alarms as defined in the safety audit and derived from the DIN ISO 14971 compliant risk management. Having performed the individual exercises a patient reported outcome form will collect symptoms during the exercise that leads to feeds in the safety audit in case of more than two reported symptoms. In the final intended use, the patients will perform the training -three to four times a week, selecting the type of training (i.e., cycling, walking, running) during any time of the day, facilitating a home-based patient-empowering environment. A 6-min walking test (6MWT) at the beginning and end of the training period measured the baseline and at the end of the program. The Quality-of-Life Questionnaire for cancer patients containing 30 questions (QLQC30) will assess quality of life before and at the end of the training period. "

5-ix) Describe use parameters
Describe use parameters (e.g., intended "doses" and optimal timing for use). Clarify what instructions or recommendations were given to the user, e.g., regarding timing, frequency, heaviness of use, if any, or was the intervention used ad libitum.

	1	2	3	4	5	
subitem not at all important	○	○	○	◉	○	essential

Auswahl löschen

Does your paper address subitem 5-ix?
Copy and paste relevant sections from the manuscript (include quotes in quotation marks "like this" to indicate direct quotes from your manuscript), or elaborate on this item by providing additional information not in the ms, or briefly explain why the item is not applicable/relevant for your study

"Eligible participants underwent a structured risk assessment and were assigned to a predefined risk score (1-6) based on the ECOG (ECOG 0=0 points, ECOG 1=1 point, ECOG>1=2 points), TUG (0 to 7 sec: 1 point; >7sec.: 2 points), RAI-C score (≤25: 0 points; >25: 2points), and hemoglobin values (Hgb>13g/dl: 0 points; ≤13g/dl: 2 points). Then, a one-time endurance exercise program following a baseline 6MWT, comprising of a 47-min interval training (five minutes warm-up, followed by alternating two minutes of high intense intervals and three minutes of low intense active rests for a total of 37 minutes, followed by a five-minute cool down) on a treadmill was performed at the Institute of Sports Science of the Goethe University Frankfurt. Participants wore one smartwatch on each wrist (one iOS and one Android software combination), which were linked to the software application on the complimentary smartphone. Four different device combinations were assessed to extend the validity of measurement: iPhone 13 + Apple Watch 7 (Apple 13), iPhone SE + Apple Watch 3 (Apple SE), Samsung A52 + Samsung Galaxy watch 4 (Samsung A52) and Google Pixel 6 + Samsung Galaxy watch 4 (Google 6). The rationale for choosing those wearables was simple. We wanted to provide the 2 major platforms used in smartphone and wearable technologies available. As google wear OS was only recently introduced, the Smartwatch 4 was the only compatible solution at thetime point of development. An extension to other wearable solutions can be performed easily later. Heart rates and distances were measured at different time points (six time points during the 6MWT and 19 time points during the interval training) by the wearables and compared to a 12-lead gold standard ECG (Custo diagnostic; Custo med GmbH, Ottobrunn, Germany) and the treadmill measurement. Participants' perceived intensity and pain during exercise were assessed using the Borg Scale and Visual and Numerical Analogue Scale (VAS, NAS). The maximum rate of oxygen consumption (VO2max) in MET (metabolic equivalent) after the 6MWT was used to determine the baseline cardiorespiratory fitness. Individual characteristics including wrist circumference, skin appendices, and skin humidity were assessed to evaluate their impact on the accuracy of measurements. As this is a fully regulated development process, the DIN ISO 13485, the MDR and all applicable legislation like the DIN ISO 27001 and the recommendations for cybersecurity implemented by the BSI in Germany have to be followed. This is done by a dedicated quality management program. On top there is an external data safety and privacy officer within the Capreolos GmbH that is in charge for this area. In brief, as the risk assessment is done by doctors on a separate app, all data are pseudonymized, and there are no personal data on the patient's app. There is only a study specific number plus a login so that the patient can only be de-pseudonymized by the clinical institutions."

5-x) Clarify the level of human involvement
Clarify the level of human involvement (care providers or health professionals, also technical assistance) in the e-intervention or as co-intervention (detail number and expertise of professionals involved, if any, as well as "type of assistance offered, the timing and frequency of the support, how it is initiated, and the medium by which the assistance is delivered". It may be necessary to distinguish between the level of human involvement required for the trial, and the level of human involvement required for a routine application outside of a RCT setting (discuss under item 21 – generalizability).

	1	2	3	4	5	
subitem not at all important	○	○	○	◉	○	essential

Auswahl löschen

Does your paper address subitem 5-x?
Copy and paste relevant sections from the manuscript (include quotes in quotation marks "like this" to indicate direct quotes from your manuscript), or elaborate on this item by providing additional information not in the ms, or briefly explain why the item is not applicable/relevant for your study

The whole intervention was supervised by a physician. The intervention was performed by the participants. Supervising physicians ensured a safe environment and collected the data, which are mentioned unter the subitem 5-ix. "In the final intended use, the patients will perform the training -three to four times a week, selecting the type of training (i.e., cycling, walking, running) during any time of the day, facilitating a home-based patient-empowering environment." This will be performed without face-to-face supervision but a remote supervision using the the App.

5-xi) Report any prompts/reminders used
Report any prompts/reminders used: Clarify if there were prompts (letters, emails, phone calls, SMS) to use the application, what triggered them, frequency etc. It may be necessary to distinguish between the level of prompts/reminders required for the trial, and the level of prompts/reminders for a routine application outside of a RCT setting (discuss under item 21 – generalizability).

	1	2	3	4	5	
subitem not at all important	○	○	◉	○	○	essential

Auswahl löschen

Does your paper address subitem 5-xi? *
Copy and paste relevant sections from the manuscript (include quotes in quotation marks "like this" to indicate direct quotes from your manuscript), or elaborate on this item by providing additional information not in the ms, or briefly explain why the item is not applicable/relevant for your study

During the trial, no prompts or reminders were used. In the final intended use, the App will remind and guide the patients to perform the exercise trainings regularly.

5-xii) Describe any co-interventions (incl. training/support)
Describe any co-interventions (incl. training/support): Clearly state any interventions that are provided in addition to the targeted eHealth intervention, as ehealth intervention may not be designed as stand-alone intervention. This includes training sessions and support [1]. It may be necessary to distinguish between the level of training required for the trial, and the level of training for a routine application outside of a RCT setting (discuss under item 21 – generalizability.

	1	2	3	4	5	
subitem not at all important	○	◉	○	○	○	essential

Auswahl löschen

Does your paper address subitem 5-xii? *
Copy and paste relevant sections from the manuscript (include quotes in quotation marks "like this" to indicate direct quotes from your manuscript), or elaborate on this item by providing additional information not in the ms, or briefly explain why the item is not applicable/relevant for your study

No co-interventions except the application of the app are planed.

6a) Completely defined pre-specified primary and secondary outcome measures, including how and when they were assessed

Does your paper address CONSORT subitem 6a? *
Copy and paste relevant sections from the manuscript (include quotes in quotation marks "like this" to indicate direct quotes from your manuscript), or elaborate on this

item by providing additional information not in the ms, or briefly explain why the item is not applicable/relevant for your study

"Safety, accuracy, and usability assessments included testing of alarm signals, and occurrence of adverse events were primary and secondary outcome measures. Heart rates and distances were measured at different time points (six time points during the 6MWT and 19 time points during the interval training) by the wearables and compared to a 12-lead gold standard ECG (Custo diagnostic; Custo med GmbH, Ottobrunn, Germany) and the treadmill measurement. Participants' perceived intensity and pain during exercise were assessed using the Borg Scale and Visual and Numerical Analogue Scale (VAS, NAS). The maximum rate of oxygen consumption (VO2max) in MET (metabolic equivalent) after the 6MWT was used to determine the baseline cardiorespiratory fitness. Individual characteristics including wrist circumference, skin appendices, and skin humidity were assessed to evaluate their impact on the accuracy of measurements. The aerobic exercise training was continuously supervised by a physician to ensure a safe environment. Secondary safety measures were based on blood samples -reported elsewhere- that were taken before and 15 minutes after the interval training (including creatinephosphatekinase, creatine-kinase-MB, lactate dehydrogenase, glucose, sodium, potassium, chloride, c-reactive protein, blood carbondioxide, lactate, and cell-free DNA (desoxy ribonucleic acid)). Moreover, awareness of participants for specific alarm signals displayed on the wearable devices, either by color or by haptic vibration when exceeding or falling below the defined heart rate ranges were assessed. "

6a-i) Online questionnaires: describe if they were validated for online use and apply CHERRIES items to describe how the questionnaires were designed/deployed

If outcomes were obtained through online questionnaires, describe if they were validated for online use and apply CHERRIES items to describe how the questionnaires were designed/deployed [9].

	1	2	3	4	5	
subitem not at all important	○	●	○	○	○	essential

Auswahl löschen

Does your paper address subitem 6a-i?

Copy and paste relevant sections from manuscript text

No online questionnaires were used.

6a-ii) Describe whether and how "use" (including intensity of use/dosage) was defined/measured/monitored

Describe whether and how "use" (including intensity of use/dosage) was defined/measured/monitored (logins, logfile analysis, etc.). Use/adoption metrics are important process outcomes that should be reported in any ehealth trial.

	1	2	3	4	5	
subitem not at all important	○	○	○	●	○	essential

Auswahl löschen

Does your paper address subitem 6a-ii?

Copy and paste relevant sections from manuscript text

Application of the app was supervised and monitored by the physician.

6a-iii) Describe whether, how, and when qualitative feedback from participants was obtained

Describe whether, how, and when qualitative feedback from participants was obtained (e.g., through emails, feedback forms, interviews, focus groups).

	1	2	3	4	5	
subitem not at all important	○	○	○	●	○	essential

Auswahl löschen

Does your paper address subitem 6a-iii?

Copy and paste relevant sections from manuscript text

Feedback from patients were obtained directly by the physician face-to-face and by using a symptom checklist provided by the app.

6b) Any changes to trial outcomes after the trial commenced, with reasons

Does your paper address CONSORT subitem 6b? *

Copy and paste relevant sections from the manuscript (include quotes in quotation marks "like this" to indicate direct quotes from your manuscript), or elaborate on this item by providing additional information not in the ms, or briefly explain why the item is not applicable/relevant for your study

There were no changes of trial outcomes after the trial commences.

7a) How sample size was determined

NPT: When applicable, details of whether and how the clustering by care provides or centers was addressed

7a-i) Describe whether and how expected attrition was taken into account when calculating the sample size

Describe whether and how expected attrition was taken into account when calculating the sample size.

	1	2	3	4	5	
subitem not at all important	○	○	○	●	○	essential

Auswahl löschen

Does your paper address subitem 7a-i?

Copy and paste relevant sections from manuscript title (include quotes in quotation marks "like this" to indicate direct quotes from your manuscript), or elaborate on this item by providing additional information not in the ms, or briefly explain why the item is not applicable/relevant for your study

"The sample size calculation was based on the assumption that a maximal irrelevant mean difference of ≤ 10bpm (beats per minute) is regarded to be acceptable. An acceptable error rate using the mean absolute percent error (MAPE) was defined to be ±10% according to recommendations. Considering a power of 80% and a one-sided alpha of 0·05, and a dropout rate of 25%, a sample size of 75 patients was required. "

7b) When applicable, explanation of any interim analyses and stopping guidelines

Does your paper address CONSORT subitem 7b? *

Copy and paste relevant sections from the manuscript (include quotes in quotation marks "like this" to indicate direct quotes from your manuscript), or elaborate on this item by providing additional information not in the ms, or briefly explain why the item is not applicable/relevant for your study

No interim analysis was performed.

8a) Method used to generate the random allocation sequence

NPT: When applicable, how care providers were allocated to each trial group

Does your paper address CONSORT subitem 8a? *

Copy and paste relevant sections from the manuscript (include quotes in quotation marks "like this" to indicate direct quotes from your manuscript), or elaborate on this item by providing additional information not in the ms, or briefly explain why the item is not applicable/relevant for your study

No random allocation was performed.

8b) Type of randomisation; details of any restriction (such as blocking and block size)

Does your paper address CONSORT subitem 8b? *

Copy and paste relevant sections from the manuscript (include quotes in quotation marks "like this" to indicate direct quotes from your manuscript), or elaborate on this item by providing additional information not in the ms, or briefly explain why the item is not applicable/relevant for your study

No randomization was performed.

9) Mechanism used to implement the random allocation sequence (such as sequentially numbered containers), describing any steps taken to conceal the sequence until interventions were assigned

Does your paper address CONSORT subitem 9? *

Copy and paste relevant sections from the manuscript (include quotes in quotation marks "like this" to indicate direct quotes from your manuscript), or elaborate on this item by providing additional information not in the ms, or briefly explain why the item is not applicable/relevant for your study

No randomization was performed.

10) Who generated the random allocation sequence, who enrolled participants, and who assigned participants to interventions

Does your paper address CONSORT subitem 10? *

Copy and paste relevant sections from the manuscript (include quotes in quotation marks "like this" to indicate direct quotes from your manuscript), or elaborate on this item by providing additional information not in the ms, or briefly explain why the item is not applicable/relevant for your study

No randomization was performed.

11a) If done, who was blinded after assignment to interventions (for example, participants, care providers, those assessing outcomes) and how

NPT: Whether or not administering co-interventions were blinded to group assignment

11a-i) Specify who was blinded, and who wasn't

Specify who was blinded, and who wasn't. Usually, in web-based trials it is not possible to blind the participants [1, 3] (this should be clearly acknowledged), but it may be possible to blind outcome assessors, those doing data analysis or those administering co-interventions (if any).

	1	2	3	4	5	
subitem not at all important	○	●	○	○	○	essential

Auswahl löschen

Does your paper address subitem 11a-i? *

Copy and paste relevant sections from the manuscript (include quotes in quotation marks "like this" to indicate direct quotes from your manuscript), or elaborate on this item by providing additional information not in the ms, or briefly explain why the item is not applicable/relevant for your study

No randomization was performed. No blinding was performed.

11a-ii) Discuss e.g., whether participants knew which intervention was the "intervention of interest" and which one was the "comparator"

Informed consent procedures (4a-ii) can create biases and certain expectations - discuss e.g., whether participants knew which intervention was the "intervention of interest" and which one was the "comparator".

	1	2	3	4	5	
subitem not at all important	○	○	●	○	○	essential

Auswahl löschen

Does your paper address subitem 11a-ii?

Copy and paste relevant sections from the manuscript (include quotes in quotation marks "like this" to indicate direct quotes from your manuscript), or elaborate on this item by providing additional information not in the ms, or briefly explain why the item is not applicable/relevant for your study

No randomization was performed. No blinding was performed.

11b) If relevant, description of the similarity of interventions

(this item is usually not relevant for ehealth trials as it refers to similarity of a placebo or sham intervention to a active medication/intervention)

Does your paper address CONSORT subitem 11b? *

Copy and paste relevant sections from the manuscript (include quotes in quotation marks "like this" to indicate direct quotes from your manuscript), or elaborate on this item by providing additional information not in the ms, or briefly explain why the item is not applicable/relevant for your study

No randomization was performed. No blinding was performed.

12a) Statistical methods used to compare groups for primary and secondary outcomes

NPT: When applicable, details of whether and how the clustering by care providers or centers was addressed

Does your paper address CONSORT subitem 12a? *

Copy and paste relevant sections from the manuscript (include quotes in quotation marks "like this" to indicate direct quotes from your manuscript), or elaborate on this item by providing additional information not in the ms, or briefly explain why the item is not applicable/relevant for your study

"Categorial variables were summarized as number (percentages), and differences between groups were compared with X2 test. Continuous variables were summarized with the Mann-Whitney U test or the Kruskal-Wallis test. Bland-Altman analysis including calculations of mean differences and 95% limits of agreement (LoA) were used to compare heart rate and distance measurements. MAPE values were calculated as the error percentage between measurements. Outliers were not removed to prevent interference with assessing the accuracy of wearable measurement. Univariate and multivariate analysis were done using a logistic regression model for assessing the impact of different individual variables (i.e., wrist circumference, skin humidity, skin appendices, risk scores) on accuracy of heart rate measurement (odds ratio (OR) ± 95% confidence intervals (95% CI) and the impact of different variables on the cardiorespiratory fitness determined by VO2max. Statistical tests were two-sided, P values ≤ ·05 were deemed statistically significant. Statistical analyses were performed with SPSS software v28.0 (IBM Corporation, Armonk, NY, USA) and R (version 4.2.2, R Studio, version 2022.07.2)."

12a-i) Imputation techniques to deal with attrition / missing values

Imputation techniques to deal with attrition / missing values: Not all participants will use the intervention/comparator as intended and attrition is typically high in ehealth trials. Specify how participants who did not use the application or dropped out from the trial were treated in the statistical analysis (a complete case analysis is strongly discouraged, and simple imputation techniques such as LOCF may also be problematic [4]).

	1	2	3	4	5	
subitem not at all important	○	●	○	○	○	essential

Auswahl löschen

Does your paper address subitem 12a-i? *

Copy and paste relevant sections from the manuscript (include quotes in quotation marks "like this" to indicate direct quotes from your manuscript), or elaborate on this item by providing additional information not in the ms, or briefly explain why the item is not applicable/relevant for your study

All participants used the interventions and were considered in the statistical analysis.

12b) Methods for additional analyses, such as subgroup analyses and adjusted analyses

Does your paper address CONSORT subitem 12b? *

Copy and paste relevant sections from the manuscript (include quotes in quotation marks "like this" to indicate direct quotes from your manuscript), or elaborate on this item by providing additional information not in the ms, or briefly explain why the item is not applicable/relevant for your study

No additional analyses was performed.

X26) REB/IRB Approval and Ethical Considerations [recommended as subheading under "Methods"] (not a CONSORT item)

X26-i) Comment on ethics committee approval

	1	2	3	4	5	
subitem not at all important	○	○	○	●	○	essential

Auswahl löschen

Does your paper address subitem X26-i?

Copy and paste relevant sections from the manuscript (include quotes in quotation marks "like this" to indicate direct quotes from your manuscript), or elaborate on this item by providing additional information not in the ms, or briefly explain why the item is not applicable/relevant for your study

"The trial protocol was approved by the institutional review board of the University Hospital Frankfurt and by the Federal Institute for Pharmaceuticals and Medical Products (BfArM, reference number 94.1.04-5660-13655) on February 7, 2022. The study was registered in the German Clinical Trial Register (DRKS 00026985) on December 21, 2021, and in the European database on medical devices (Eudamed, CIV-21-07-0307311). "

x26-ii) Outline informed consent procedures

Outline informed consent procedures e.g., if consent was obtained offline or online (how? Checkbox, etc.?), and what information was provided (see 4a-ii). See [6] for some items to be included in informed consent documents.

	1	2	3	4	5	
subitem not at all important	○	○	○	●	○	essential

Auswahl löschen

Does your paper address subitem X26-ii?

Copy and paste relevant sections from the manuscript (include quotes in quotation marks "like this" to indicate direct quotes from your manuscript), or elaborate on this item by providing additional information not in the ms, or briefly explain why the item is not applicable/relevant for your study

"All participants provided written informed consent." This was obtained online in the clinic by the study doctors.

X26-iii) Safety and security procedures

Safety and security procedures, incl. privacy considerations, and any steps taken to reduce the likelihood or detection of harm (e.g., education and training, availability of a hotline)

	1	2	3	4	5	
subitem not at all important	○	○	○	●	○	essential

Auswahl löschen

Does your paper address subitem X26-iii?

Copy and paste relevant sections from the manuscript (include quotes in quotation marks "like this" to indicate direct quotes from your manuscript), or elaborate on this item by providing additional information not in the ms, or briefly explain why the item is not applicable/relevant for your study

"As this is a fully regulated development process, the DIN ISO 13485, the MDR and all applicable legislation like the DIN ISO 27001 and the recommendations for cybersecurity implemented by the BSI in Germany have to be followed. This is done by a dedicated quality management program. On top there is an external data safety and privacy officer within the Capreolos GmbH that is in charge for this area. In brief, as the risk assessment is done by doctors on a separate app, all data are pseudonymized, and there are no personal data on the patient's app. There is only a study specific number plus a login so that the patient can only be de-pseudonymized by the clinical institutions."

RESULTS

13a) For each group, the numbers of participants who were randomly assigned, received intended treatment, and were analysed for the primary outcome

NPT: The number of care providers or centers performing the intervention in each group and the number of patients treated by each care provider in each center

Does your paper address CONSORT subitem 13a? *

Copy and paste relevant sections from the manuscript (include quotes in quotation marks "like this" to indicate direct quotes from your manuscript), or elaborate on this item by providing additional information not in the ms, or briefly explain why the item is not applicable/relevant for your study

No randomization was performed.

13b) For each group, losses and exclusions after randomisation, together with reasons

Does your paper address CONSORT subitem 13b? (NOTE: Preferably, this is shown in a CONSORT flow diagram) *

Copy and paste relevant sections from the manuscript (include quotes in quotation marks "like this" to indicate direct quotes from your manuscript), or elaborate on this item by providing additional information not in the ms, or briefly explain why the item is not applicable/relevant for your study

No randomization was performed.

13b-i) Attrition diagram

Strongly recommended: An attrition diagram (e.g., proportion of participants still logging in or using the intervention/comparator in each group plotted over time, similar to a survival curve) or other figures or tables demonstrating usage/dose/engagement.

	1	2	3	4	5	
subitem not at all important	○	●	○	○	○	essential

Auswahl löschen

Does your paper address subitem 13b-i?

Copy and paste relevant sections from the manuscript or cite the figure number if applicable (include quotes in quotation marks "like this" to indicate direct quotes from your manuscript), or elaborate on this item by providing additional information not in the ms, or briefly explain why the item is not applicable/relevant for your study

Patients underwent intervention during a a one-time endurance exercise program. No usage of the application after the exercise was planned.

14a) Dates defining the periods of recruitment and follow-up

Does your paper address CONSORT subitem 14a? *

Copy and paste relevant sections from the manuscript (include quotes in quotation marks "like this" to indicate direct quotes from your manuscript), or elaborate on this item by providing additional information not in the ms, or briefly explain why the item is not applicable/relevant for your study

"Of the 77 participants recruited, 75 were included. Two individuals were excluded on the training day after double-checking the exclusion criteria, because they gave misleading information during the first interview. Finally, a total of 75 participants with 150 wearables-smartphone combinations were analyzed, of whom no one was lost to follow-up. "

14a-i) Indicate if critical "secular events" fell into the study period

Indicate if critical "secular events" fell into the study period, e.g., significant changes in Internet resources available or "changes in computer hardware or Internet delivery resources"

	1	2	3	4	5	
subitem not at all important	○	●	○	○	○	essential

Auswahl löschen

Does your paper address subitem 14a-i?

Copy and paste relevant sections from the manuscript (include quotes in quotation marks "like this" to indicate direct quotes from your manuscript), or elaborate on this item by providing additional information not in the ms, or briefly explain why the item is not applicable/relevant for your study

No secular events occurred.

14b) Why the trial ended or was stopped (early)

Does your paper address CONSORT subitem 14b? *

Copy and paste relevant sections from the manuscript (include quotes in quotation marks "like this" to indicate direct quotes from your manuscript), or elaborate on this item by providing additional information not in the ms, or briefly explain why the item is not applicable/relevant for your study

The trial did not ended earlier than planned.

15) A table showing baseline demographic and clinical characteristics for each group

NPT: When applicable, a description of care providers (case volume, qualification, expertise, etc.) and centers (volume) in each group

Does your paper address CONSORT subitem 15? *

Copy and paste relevant sections from the manuscript (include quotes in quotation marks "like this" to indicate direct quotes from your manuscript), or elaborate on this item by providing additional information not in the ms, or briefly explain why the item is not applicable/relevant for your study

A detailed table is provided in the manuscript.

15-i) Report demographics associated with digital divide issues

In ehealth trials it is particularly important to report demographics associated with digital divide issues, such as age, education, gender, social-economic status, computer/Internet/ehealth literacy of the participants, if known.

	1	2	3	4	5	
subitem not at all important	○	○	○	●	○	essential

Auswahl löschen

Does your paper address subitem 15-i? *

Copy and paste relevant sections from the manuscript (include quotes in quotation marks "like this" to indicate direct quotes from your manuscript), or elaborate on this item by providing additional information not in the ms, or briefly explain why the item is not applicable/relevant for your study

""The study population was divided into three subgroups, comprising 26 (35%) participants with a Risk Score 0, 38 (51%) with a Risk Score 1-3, and 11 (14%) with a Risk Score 4-6 (Table 1). The median age was significantly higher in the Risk Score 4-6 group (63 vs 46 and 57, P = ·01), consisting of significantly more male participants (9 [82%] vs 12 [46%] and 29 [76%], P = ·02). No differences were found in terms of BMI or smoking. Importantly, high, and medium risk patients more often suffered from chronic cardiovascular or pulmonary disease compared to the other groups. High-risk patients were most commonly from the Visceral (5 [46%]) and Thoracic Surgical specialties (1 [9%]) undergoing HPB and lung resections, while urological patients (23 [61%]) were more often assigned to the medium risk group, scheduled for radical prostatectomy and nephrectomy. Low-risk patients predominantly comprised Gynecological (4 [15%]) and Vascular Surgical (3 [12%]) patients undergoing salpingo-/oophorectomy, hysterectomy, or carotid endarterectomy. Consistently to the cardiopulmonary comorbidities, structured assessment of risk factors depicted significantly higher percentage of RAI scores ≥ 26 for the high-risk group (5 [47%] vs 7 [18%] and 0 [0%], P < ·001) and TUG-scores: 7-11 seconds (10 [91%] vs. 18 [47%] and 8 [31%], P = ·004), while there were no differences regarding ECOG, and hemoglobin. "

16) For each group, number of participants (denominator) included in each analysis and whether the analysis was by original assigned groups

16-i) Report multiple "denominators" and provide definitions

Report multiple "denominators" and provide definitions: Report N's (and effect sizes) "across a range of study participation [and use] thresholds" [1], e.g., N exposed, N consented, N used more than x times, N used more than y weeks, N participants "used" the intervention/comparator at specific pre-defined time points of interest (in absolute and relative numbers per group). Always clearly define "use" of the intervention.

	1	2	3	4	5	
subitem not at all important	○	○	●	○	○	essential

Auswahl löschen

Does your paper address subitem 16-i? *

Copy and paste relevant sections from the manuscript (include quotes in quotation marks "like this" to indicate direct quotes from your manuscript), or elaborate on this item by providing additional information not in the ms, or briefly explain why the item is not applicable/relevant for your study

All participants used the intervention once during the exercise program. No usage of the intervention afterwards was planned.

16-ii) Primary analysis should be intent-to-treat

Primary analysis should be intent-to-treat, secondary analyses could include comparing

only “users”, with the appropriate caveats that this is no longer a randomized sample (see 18-i).

subitem not at all important ○ 1 ◉ 2 ○ 3 ○ 4 ○ 5 essential

Auswahl löschen

Does your paper address subitem 16-ii?
Copy and paste relevant sections from the manuscript (include quotes in quotation marks "like this" to indicate direct quotes from your manuscript), or elaborate on this item by providing additional information not in the ms, or briefly explain why the item is not applicable/relevant for your study

No randomization was performed.

17a) For each primary and secondary outcome, results for each group, and the estimated effect size and its precision (such as 95% confidence interval)

Does your paper address CONSORT subitem 17a? *
Copy and paste relevant sections from the manuscript (include quotes in quotation marks "like this" to indicate direct quotes from your manuscript), or elaborate on this item by providing additional information not in the ms, or briefly explain why the item is not applicable/relevant for your study

"This analysis presents the first pilot study focusing on the validity and safety assessment of a risk-stratified prehabilitation program for patients undergoing major elective surgery using an mHealth App, which is linked to wearable devices providing self- and remote monitoring of physical performance. The heart rate measurement was below the recommended threshold with a MAPE ≤ 5% showing excellent accuracy. Laboratory analysis and assessment of AEs indicated that the individually tailored exercise training can be safely performed in a supervised setting and that a remote testing of The Prehab App in a multicentric randomized controlled trial is justifiable. "

17a-i) Presentation of process outcomes such as metrics of use and intensity of use
In addition to primary/secondary (clinical) outcomes, the presentation of process outcomes such as metrics of use and intensity of use (dose, exposure) and their operational definitions is critical. This does not only refer to metrics of attrition (13-b) (often a binary variable), but also to more continuous exposure metrics such as “average session length”. These must be accompanied by a technical description how a metric like a “session” is defined (e.g., timeout after idle time) [1] (report under item 6a).

subitem not at all important ○ 1 ○ 2 ◉ 3 ○ 4 ○ 5 essential

Auswahl löschen

Does your paper address subitem 17a-i?
Copy and paste relevant sections from the manuscript (include quotes in quotation marks "like this" to indicate direct quotes from your manuscript), or elaborate on this item by providing additional information not in the ms, or briefly explain why the item is not applicable/relevant for your study

All participants used the application only once during the exercise for the same intensity and time.

17b) For binary outcomes, presentation of both absolute and relative effect sizes is recommended

Does your paper address CONSORT subitem 17b? *
Copy and paste relevant sections from the manuscript (include quotes in quotation marks "like this" to indicate direct quotes from your manuscript), or elaborate on this item by providing additional information not in the ms, or briefly explain why the item is not applicable/relevant for your study

No randomization was performed. All participants used the application in the same way.

18) Results of any other analyses performed, including subgroup analyses and adjusted analyses, distinguishing pre-specified from exploratory

Does your paper address CONSORT subitem 18? *
Copy and paste relevant sections from the manuscript (include quotes in quotation marks "like this" to indicate direct quotes from your manuscript), or elaborate on this item by providing additional information not in the ms, or briefly explain why the item is not applicable/relevant for your study

No randomization was performed. All participants used the application in the same way.

18-i) Subgroup analysis of comparing only users
A subgroup analysis of comparing only users is not uncommon in ehealth trials, but if done, it must be stressed that this is a self-selected sample and no longer an unbiased sample from a randomized trial (see 16-iii).

subitem not at all important ○ 1 ◉ 2 ○ 3 ○ 4 ○ 5 essential

Auswahl löschen

Does your paper address subitem 18-i?
Copy and paste relevant sections from the manuscript (include quotes in quotation marks "like this" to indicate direct quotes from your manuscript), or elaborate on this item by providing additional information not in the ms, or briefly explain why the item is not applicable/relevant for your study

No randomization was performed. All participants used the application in the same way.

19) All important harms or unintended effects in each group
(for specific guidance see CONSORT for harms)

Does your paper address CONSORT subitem 19? *
Copy and paste relevant sections from the manuscript (include quotes in quotation marks "like this" to indicate direct quotes from your manuscript), or elaborate on this item by providing additional information not in the ms, or briefly explain why the item is not applicable/relevant for your study

"The safety analysis of laboratory values, alarm settings of the App and occurrence of adverse events was comprised (AE) (Table 4). Lab analysis did not reveal severe deviations of blood parameters which remained within clinical limits. A detailed analysis from an exercise physiologists view will be reported elsewhere. Awareness of alarm signals displayed on the wearables during the interval training were realized with a mean (SD) of 13·5 (6·2) seconds. In total, 29 (39%) experienced an AE, of which 12 (16%) occurred during the interval training, comprising pain (5 [42%]), skin irritation through ECG electrodes (redness or vesicles) (2 [17%]), dizziness (2 [17%]), shortness of breath (2 [17%]), and fatigue (1 [8%]). During the follow-up at day 7, three (20%) participants still reported about pain and 12 (80%) indicated skin irritations, which remained until the follow-up at day 30 in two cases. Notably, 14 events were related to the certified ECG (electrodes) which had nothing to do with the use of the software but needed to be reported for regulatory reasons. There were no serious adverse events or device deficiencies that put the patient at an unacceptable risk. All AEs were periodically reported to the BfArM according to the regulations of the MDR and the MPDG, and no AE lasted longer than 30 days. No serious adverse event was reported during the trial and no patient died until follow-up."^

19-i) Include privacy breaches, technical problems
Include privacy breaches, technical problems. This does not only include physical “harm” to participants, but also incidents such as perceived or real privacy breaches [1], technical problems, and other unexpected/unintended incidents. “Unintended effects” also includes unintended positive effects [2].

subitem not at all important ○ 1 ◉ 2 ○ 3 ○ 4 ○ 5 essential

Auswahl löschen

Does your paper address subitem 19-i?
Copy and paste relevant sections from the manuscript (include quotes in quotation marks "like this" to indicate direct quotes from your manuscript), or elaborate on this item by providing additional information not in the ms, or briefly explain why the item is not applicable/relevant for your study

No technical problems with the App occurred.

19-ii) Include qualitative feedback from participants or observations from staff/researchers
Include qualitative feedback from participants or observations from staff/researchers, if available, on strengths and shortcomings of the application, especially if they point to unintended/unexpected effects or uses. This includes (if available) reasons for why people did or did not use the application as intended by the developers.

subitem not at all important ○ 1 ○ 2 ◉ 3 ○ 4 ○ 5 essential

Auswahl löschen

Does your paper address subitem 19-ii?
Copy and paste relevant sections from the manuscript (include quotes in quotation marks "like this" to indicate direct quotes from your manuscript), or elaborate on this item by providing additional information not in the ms, or briefly explain why the item is not applicable/relevant for your study

All adverse events are described in subitem 19.

DISCUSSION

22) Interpretation consistent with results, balancing benefits and harms, and considering other relevant evidence
NPT: In addition, take into account the choice of the comparator, lack of or partial blinding, and unequal expertise of care providers or centers in each group

22-i) Restate study questions and summarize the answers suggested by the data, starting with primary outcomes and process outcomes (use)
Restate study questions and summarize the answers suggested by the data, starting with primary outcomes and process outcomes (use).

subitem not at all important ○ 1 ○ 2 ○ 3 ◉ 4 ○ 5 essential

Auswahl löschen

Does your paper address subitem 22-i? *
Copy and paste relevant sections from the manuscript (include quotes in quotation marks "like this" to indicate direct quotes from your manuscript), or elaborate on this item by providing additional information not in the ms, or briefly explain why the item is not applicable/relevant for your study

"To the best of our knowledge, this is the first prospective trial report assessing the validity and safety of a risk-adjusted, App-based prehabilitation program using wearables for heart rate and physical activity monitoring. The analysis revealed a high accuracy of wearable heart rate measurement with a mean difference ≤5 bpm and a MAPE ≤5%, while distance measurement exhibited larger variations (mean difference ≤ 209 bpm, MAPE ≤ 31%), which can be explained through an impaired detection of patient’s wrist movement during the exercise training on the treadmill and the lack of Global Positioning System (GPS) trackers."

22-ii) Highlight unanswered new questions, suggest future research
Highlight unanswered new questions, suggest future research.

subitem not at all important ○ 1 ○ 2 ○ 3 ◉ 4 ○ 5 essential

Auswahl löschen

Does your paper address subitem 22-ii?
Copy and paste relevant sections from the manuscript (include quotes in quotation marks "like this" to indicate direct quotes from your manuscript), or elaborate on this item by providing additional information not in the ms, or briefly explain why the item is not applicable/relevant for your study

"At this stage it seems, that a resource intensive prehabilitation program only works in clinical trials due to the enormous infrastructure requirements and costs associated with it including travelling of patients over weeks which seems hardly feasible which seems to be a real chance for a well guided and safe remote approach, that additionally fosters patient empowerment "

20) Trial limitations, addressing sources of potential bias, imprecision, and, if relevant, multiplicity of analyses

20-i) Typical limitations in ehealth trials
Typical limitations in ehealth trials: Participants in ehealth trials are rarely blinded. Ehealth trials often look at a multiplicity of outcomes, increasing risk for a Type I error. Discuss biases due to non-use of the intervention/usability issues, biases through informed consent procedures, unexpected events.

subitem not at all important ○ 1 ○ 2 ◉ 3 ○ 4 ○ 5 essential

Auswahl löschen

Does your paper address subitem 20-i? *
Copy and paste relevant sections from the manuscript (include quotes in quotation marks "like this" to indicate direct quotes from your manuscript), or elaborate on this item by providing additional information not in the ms, or briefly explain why the item is not applicable/relevant for your study

"The study has strengths and weaknesses that will be addressed in the following. The strength of the study is that we follow a strict regulatory framework backed by a dedicated quality and risk management. All criteria assessed here were derived from risk management. This explains the stepwise approach from a first in men pilot character in health students and patients with the intended use. The study was split up in a pure usability part and evaluation of the risk assessment which will be reported elsewhere. Another strength is the use of evidence-based risk calculation tools. The RAI score seemed the most suitable and easy to use score that did not require additional tools to assess. This one combined with the TUG and the ECOG were most suitable to the requirements used in a digital solution. Furthermore, it is currently highlighted as the so called surgical pause by the Americal College of Surgeons".
"A weakness of the study may be that it is not a randomized controlled trial. However, as we create evidence in a young area of clinical medicine safety and validity was the first stepwise approach followed by a multicentric clinical trial that is currently launched. Comparability at this stage of remote prehabilitation is very difficult as there are different methods to perform the remote prehabilitation. Van der Velde et al. performed a pilot randomized controlled trial in which they used a smartphone based app to achieve behavioral changes before surgery. They found self reported changes in alcohol use, nicotine cessation and an increase in self-reported exercising. Wang and coworkers describe their development of an app to foster prehabilitation and assessed usability. They found a promising usability result with their device but without showing any efficacy data so far. Whilst the idea of the two beforehand mentioned was only comparable to ours, the solutions lacked a supervision of what happened during the exercises, and it is unclear whether the patients had a light or moderate to vigorous exercise which has much larger effects. Haveman et al. follow a similar approach as we do, however doing it differently. They tracked peoples activities and interpreted the increase of HR and step counts 3 days before surgery as an increased physical activity without a specific readout from the data assesse. This shows that remote prehabilitation seems feasible, is explored and may be a hot topic for the next years as outlined in various arguments and discussion points before."

21) Generalisability (external validity, applicability) of the trial findings
NPT: External validity of the trial findings according to the intervention, comparators, patients, and care providers or centers involved in the trial

21-i) Generalizability to other populations
Generalizability to other populations: In particular, discuss generalizability to a general Internet population, outside of a RCT setting, and general patient population, including applicability of the study results for other organizations

subitem not at all important ○ 1 ○ 2 ○ 3 ◉ 4 ○ 5 essential

Auswahl löschen

Does your paper address subitem 21-i?
Copy and paste relevant sections from the manuscript (include quotes in quotation marks "like this" to indicate direct quotes from your manuscript), or elaborate on this item by providing additional information not in the ms, or briefly explain why the item is not applicable/relevant for your study

This is a pilot trial, which does not ensure a generalizability to a general population. Further studies, including a RCT are required, to allow a generalizability.

21-ii) Discuss if there were elements in the RCT that would be different in a routine application setting
Discuss if there were elements in the RCT that would be different in a routine application setting (e.g., prompts/reminders, more human involvement, training sessions or other co-interventions) and what impact the omission of these elements could have on use, adoption, or outcomes if the intervention is applied outside of a RCT setting.

subitem not at all important ○ 1 ◉ 2 ○ 3 ○ 4 ○ 5 essential

Auswahl löschen

Does your paper address subitem 21-ii?
Copy and paste relevant sections from the manuscript (include quotes in quotation marks "like this" to indicate direct quotes from your manuscript), or elaborate on this item by providing additional information not in the ms, or briefly explain why the item is not applicable/relevant for your study

"In the final intended use, the patients will perform the training -three to four times a week, selecting the type of training (i.e., cycling, walking, running) during any time of the day, facilitating a home-based patient-empowering environment. "

OTHER INFORMATION

23) Registration number and name of trial registry

Does your paper address CONSORT subitem 23? *
Copy and paste relevant sections from the manuscript (include quotes in quotation marks "like this" to indicate direct quotes from your manuscript), or elaborate on this item by providing additional information not in the ms, or briefly explain why the item is not applicable/relevant for your study

"The study was registered in the German Clinical Trial Register (DRKS 00026985) on December 21, 2021, and in the European database on medical devices (Eudamed, CIV-21-07-0307311). "

24) Where the full trial protocol can be accessed, if available

Does your paper address CONSORT subitem 24? *
Cite a Multimedia Appendix, other reference, or copy and paste relevant sections from the manuscript (include quotes in quotation marks "like this" to indicate direct quotes from your manuscript), or elaborate on this item by providing additional information not in the ms, or briefly explain why the item is not applicable/relevant for your study

A study protocol of the trial has been published previously.
"Faqar-Uz-Zaman SF, Sliwinski S, Detemble C, et al. Study protocol for a pilot trial analysing the usability, validity and safety of an interventional health app programme for the structured prehabilitation of patients before major surgical interventions: the PROTEGO MAXIMA trial. BMJ Open. 2023;13:e069394"

25) Sources of funding and other support (such as supply of drugs), role of funders

Does your paper address CONSORT subitem 25? *
Copy and paste relevant sections from the manuscript (include quotes in quotation marks "like this" to indicate direct quotes from your manuscript), or elaborate on this item by providing additional information not in the ms, or briefly explain why the item is not applicable/relevant for your study

"The clinical trial was funded by the Else-Kroener-Fresenius Stiftung in the translational research program (number: 2021_EKTP10). The funder had no role in study design, data collection, data analysis, data interpretation, or the writing of the manuscript."

X27) Conflicts of Interest (not a CONSORT item)

X27-i) State the relation of the study team towards the system being evaluated
In addition to the usual declaration of interests (financial or otherwise), also state the relation of the study team towards the system being evaluated, i.e., state if the authors/evaluators are distinct from or identical with the developers/sponsors of the intervention.

subitem not at all important ○ 1 ○ 2 ◉ 3 ○ 4 ○ 5 essential

Auswahl löschen

Does your paper address subitem X27-i?
Copy and paste relevant sections from the manuscript (include quotes in quotation marks "like this" to indicate direct quotes from your manuscript), or elaborate on this item by providing additional information not in the ms, or briefly explain why the item is not applicable/relevant for your study

There are no conflicts of interest.

About the CONSORT EHEALTH checklist

As a result of using this checklist, did you make changes in your manuscript? *

- ○ yes, major changes
- ○ yes, minor changes
- ◉ no

What were the most important changes you made as a result of using this checklist?

No changes were made in the manuscript.

How much time did you spend on going through the checklist INCLUDING making changes in your manuscript *

No changes were made in the manuscript.

As a result of using this checklist, do you think your manuscript has improved? *

- ◉ yes
- ○ no
- ○ Sonstiges:

Would you like to become involved in the CONSORT EHEALTH group?
This would involve for example becoming involved in participating in a workshop and writing an "Explanation and Elaboration" document

- ○ yes
- ◉ no
- ○ Sonstiges:

Auswahl löschen

Any other comments or questions on CONSORT EHEALTH

Meine Antwort

STOP - Save this form as PDF before you click submit
To generate a record that you filled in this form, we recommend to generate a PDF of this page (on a Mac, simply select "print" and then select "print as PDF") before you submit it.

When you submit your (revised) paper to JMIR, please upload the PDF as supplementary file.

Don't worry if some text in the textboxes is cut off, as we still have the complete information in our database. Thank you!

Final step: Click submit !
Click submit so we have your answers in our database!

Senden Alle Eingaben löschen

Geben Sie niemals Passwörter über Google Formulare weiter.

Dieser Inhalt wurde nicht von Google erstellt und wird von Google auch nicht unterstützt. Missbrauch melden - Nutzungsbedingungen - Datenschutzerklärung

Google Formulare
